# Questionnaires Assessing Adolescents’ Self-Concept, Self-Perception, Physical Activity and Lifestyle: A Systematic Review

**DOI:** 10.3390/children9010091

**Published:** 2022-01-10

**Authors:** Natacha Palenzuela-Luis, Gonzalo Duarte-Clíments, Juan Gómez-Salgado, José Ángel Rodríguez-Gómez, María Begoña Sánchez-Gómez

**Affiliations:** 1 Department of Nursing, University Hospital of Canarias, 38320 Santa Cruz de Tenerife, Spain; natacha_pl@hotmail.com; 2 Department of Nursing, University School of Nursing, Candelaria N.S. University Hospital, University of La Laguna, 38010 Santa Cruz de Tenerife, Spain; extgduartcl@ull.edu.es (G.D.-C.); begonasanchez@gmail.com (M.B.S.-G.); 3 Department of Sociology, Social Work and Public Health, Faculty of Labour Sciences, University of Huelva, 21007 Huelva, Spain; 4 Safety and Health Postgraduate Program, Espíritu Santo University, Guayaquil 091650, Ecuador; 5 Department of Nursing, Faculty of Health Sciences, University of La Laguna, 38071 Santa Cruz de Tenerife, Spain; jarogo@ull.es

**Keywords:** adolescent, self-concept, self-perception, exercise, lifestyle, questionnaires

## Abstract

Introduction: Adolescence is considered a fundamental time to promote change. During this time, young people consolidate their social and individual identity. By influencing positive changes, chronic diseases can be avoided, delayed or modified in the future. The use of valid and reliable questionnaires is an optimal resource for gathering information and thus useful for this study. Objectives: The objectives of the study were to: (1). identify the questionnaires that assess self-esteem/self-concept, self-perception, physical exercise and lifestyle of adolescents; (2). analyse the psychometric measures of the questionnaires used to assess the self-esteem/self-concept, self-perception, physical exercise and lifestyle of adolescents; and (3). determine which questionnaires are the most reliable and valid for assessing the self-esteem/self-concept, self-perception, physical exercise and lifestyle of adolescents. Method: A bibliographic search was carried out in the following databases: Virtual Health Library, Cochrane, Medline, Cuiden, Scielo, Dialnet, PubMed and Ministry of Health, Consumption and Social Welfare following the PICO method. The recommendations of the PRISMA statement were followed. Results: A total of 71 scientific articles were collected. Within the self-perception/self-concept questionnaires, the Rosenberg Self-Esteem Scale stands out for being an optimal and widely used resource in adolescents. Regarding the questionnaires that evaluate self-perception, the General Health Questionnaire is the most used; it is used in numerous national health surveys in different countries. The Physical Activity Questionnaire for Adolescents ranks first with respect to the rest of the tools. It is a widely used resource internationally and provides enough information on the physical activity carried out by the subject in a given week. Although there are several questionnaires that measure lifestyle, the Health Behavior in School-aged Children instrument was selected. This instrument is at the European level and involves the collaboration of 48 countries and allows us to compare the lifestyle habits of adolescents from different countries. Discussion: The questionnaire that stands out in the assessment of self-esteem/self-concept is the Rosenberg Self-Esteem Scale. The General Health Questionnaire has been selected as the best tool for assessing self-perception. To measure physical exercise, the Physical Activity Questionnaire for Adolescents is identified as the ideal instrument because it is widely used and can be completed quickly. Regarding lifestyle, the Health Behavior in School aged Children is shown to be an effective instrument in assessing lifestyle.

## 1. Introduction

Adolescence is the period of time between the end of puberty and the beginning of adulthood. At this time, individual and social identity is consolidated. Young people experience major physical, psychological and behavioral changes. This implies an identity crisis, contrasting emotions and new social relationships. Therefore, it is considered a susceptible stage that affects individual self-concept and self-perception. Indeed, adolescents’ lifestyles are strongly associated with body dissatisfaction, this relation being mediated by physical activity and body image; thus, a healthy lifestyle increases the probability of having better fitness and fatness, which can indirectly cause a decrease in body dissatisfaction [1]. Various studies indicate that it is necessary to take care of the physical, psychological and social health of young people, since the attitudes acquired and internalised during this period have a major impact on adulthood [2,3,4]. Proper prevention and health promotion during this period could prevent, delay or modify chronic diseases in adulthood, such as low self-concept, low self-perception, sedentary lifestyle or eating problems [5].

The terms self-esteem and self-concept are related. Self-esteem is a person’s view of his or her potential, self-efficacy, self-worth and self-definition [6,7,8]. Self-concept is the perception that the subject develops about him/herself, i.e., what he/she can achieve, what others believe he/she is and how he/she intends to be [2,9]. Both concepts contribute to the construction of the adolescent’s identity. They influence the way in which subjects think, behave and relate to their environment [8,10]. It is therefore necessary to foster good self-esteem and self-concept among adolescents. These terms take on great importance in young people since having a low self-concept predisposes the adolescent to develop psychiatric pathologies, such as depression, anxiety or feelings of inferiority. [6,7,8,9,10].

Self-perception of health is related to physical activity, level of income, consumption of toxic substances, sex, presence of a disease, level of social participation, etc. [11]. It is defined as a set of internal conscious and organised self-concepts in an individual. Interventions in the way in which the adolescent perceives oneself is necessary since it significantly influences the physical and psychological state. Thus, those young people who feel loneliness and dissatisfaction with their lives often develop aggressive behaviors. [12,13,14].

Physical exercise is necessary for the proper development of the individual. The practice of physical exercise is instilled from childhood since the benefits it generates in people’s health have been demonstrated. If its practice is instilled from an early age, it is practically certain that healthy habits will be acquired and will last over time [15,16]. During adolescence, the practice of physical exercise is diminished on many occasions as with women who devote less time to its practice. Influencing the young person to stay active is necessary for their physical and mental health. It is related to better self-concept and self-esteem and contributes to better academic performance. For this reason, including questionnaires assessing physical activity in adolescents in health surveys is considered logical [17,18].

Assessing the lifestyle habits of adolescents is necessary to understand their health management. Harmful attitudes in the lifestyle of adolescents should be examined and modified, facilitating corrective measures to prevent the acquisition of harmful lifestyle habits. In particular, this applies to nutritional habits, as there are specific requirements during this period, and it is necessary to ensure adequate nutrition. For example, although breakfast is one of the most important meals of the day, its omission or improper preparation is a common practice in this age group [3,19]. In recent years, eating disorders (EDs) have increased, leading to complications that manifest themselves in adulthood [20,21]. It is therefore necessary to have an impact on the healthy lifestyle of young people, as the behaviours acquired in this period determine the habits of adults [9].

From the above, two main ideas can be extracted: on the one hand, the importance of assessing the habits of adolescents and their impact in terms of self-concept and self-perception; and on the other, the need for valid and reliable measurement instruments for these aspects [1,2,3,4].

In order to care for adolescents, it is necessary to know how they manage their health. The use of questionnaires as a research tool is a useful resource for the assessment of adolescents. These instruments allow us to gather information on psychological characteristics, lifestyle and the adolescent’s immediate environment. To do so, they must meet certain psychometric characteristics: they must be reliable and valid, written in a coherent and organised way, sequenced and structured by means of appropriate planning. The tools collected in this systematic review can be grouped into four categories: self-concept, self-perception of health, physical activity and lifestyle.

There is a relationship between self-esteem and self-concept, as well as between self-perception and physical activity. Leading a healthy lifestyle favours improvements in self-concept and self-perception [3,6,7,8,11,19]. Knowing the tools for measuring these concepts means an improvement not only for the individual, who would be able to improve their health habits, but also for the professional who assesses them, because they will have a valid and reliable instrument allowing them to establish corrective measures for the individual. Improving these concepts in adolescence is considered fundamental. Establishing changes in the lifestyle of young people prevents the development of physical and psychological problems, and chronic diseases in adulthood [1,2,3,4,9,11,19].

The need for this study arises because, despite the amount of information adolescents receive about healthy lifestyle habits, the number of overweight young people continues to increase in developed countries. It also highlights the increase in psychiatric pathologies, such as anxiety or depression, among the youth population. Knowing the adolescent is necessary to establish corrective measures that favour the adequate transition of the young person to the adult stage. Identifying the tools that evaluate the current state of self-concept, self-perception, physical exercise and lifestyle helps to create an optimal approach for the adolescent. This study provides a valid and reliable review that helps professionals in making favourable changes in the lifestyle of young people [9,11,19].

The aim of this study is to review the most widely used questionnaires at international level to assess self-concept, self-perception, physical activity, and lifestyle among adolescents. Specifically, this objective can be subdivided into to analyse the psychometric properties of the questionnaires used for the assessment of self-concept, self-perception, physical activity, and lifestyle of adolescents; and to determine which questionnaires are the most reliable and valid for the assessment of self-concept, self-perception, physical activity, and lifestyle of adolescents.

## 2. Materials and Methods

The recommendations of the PRISMA statement were followed in this systematic review (Figure S1). This is an up-to-date guide for conducting systematic reviews in a correct and organised way [22].

### 2.1. Eligibility Criteria

The inclusion criteria were questionnaires applicable to adolescence; questionnaires valid for persons in their university years; and questionnaires widely used in national and international articles assessing self-concept, self-perception, physical exercise and lifestyle in adolescents. As exclusion criteria, articles that did not exceed 8 on the CASPe score were excluded; publications with no scientific basis, with a non-representative sample, or where the representativeness was not stated in the description of the sample, articles in which statistical significance was not stated if applicable or whose results were not statistically significant, and questionnaires with Cronbach’s alpha lower than 0.75 were also excluded.

### 2.2. Sources of Information

All the questionnaires found in the literature search that meet the eligibility criteria were compiled. The PICO structure was used: patient/population: adolescents and university students; intervention: instruments and questionnaires; comparator: test; outcome: self-concept, self-perception, physical exercise and lifestyle.

The date of the last search was 1 November 2021.

### 2.3. Search Strategy

A search was carried out in the following scientific databases (DB): Virtual Health Library (VHL), Cochrane, Medline, Cuiden, Scielo, Dialnet, PubMed, and the website of the Ministry of Health, Consumer Affairs and Social Welfare (Table 1). This was expanded by means of a reference search when appropriate. The search limits or filters used were open access articles with full text available, articles published in the last 5 years, studies conducted in Spanish, English and Portuguese.

### 2.4. Study Selection Process

Studies were selected using the CASPe [23] system for critical reading. In this systematic review, the level accepted as valid for critical reading screening was 8 points. The quality assessment was completed independently by peers and discrepancies were resolved by discussion and consensus among the research group. The degree of recommendation of each article was also assessed using the Grading of Recommendations, Assessment, Development and Evaluation (GRADE) approach [24]. Four categories were established: high, moderate, low or very low. Articles with evidence of high and/or moderate quality rating were selected (Table S1). The psychometric quality of the instruments was assessed with the COSMIN [25] tool for all the questionnaires in the study (Table S2). The quality of the instruments in outcome measurement was therefore assessed with an 11-step procedure. No automation tools were used.

### 2.5. Data Collection Process

Data was collected qualitatively by all researchers, by pairs, with discrepancies identified and qualitatively extracted in a consensual way to produce the data list. No automation tools were used.

### 2.6. Data List

In order to structure the collected data, results compatible with all the measurement scales found were searched for and classified by considering self-esteem/self-concept questionnaires, self-perception questionnaires, physical activity questionnaires and lifestyle questionnaires.

### 2.7. Assessment of the Risk of Bias in Individual Studies

In the critical reading, attention was paid to this aspect, looking at their internal structure, validity and reliability. The study population, variables, interventions used, results and grade of recommendation were analysed using the GRADE [24] approach (Table S1).

### 2.8. Synthesis Methods

All the studies identified with a moderate or high level were compiled, based on the evaluation with the GRADE [24] approach. The most widely used, valid, reliable questionnaires that meet the characteristics of assessment of self-concept, self-perception, physical exercise and lifestyle in adolescents according to the COSMIN [25] protocols were analysed and selected. Table S2 shows the questionnaires collected in this systematic review and those that have been selected for their optimal characteristics in the assessment of the aforementioned concepts.

### 2.9. Assessment of Publication Bias

Once this systematic review was completed, the CASPe [23] system was applied to self-check for possible methodological bias, giving a score of 10/10 points for systematic reviews and 11/11 for case and control studies. The peer review was evaluated among the investigators in pairs to check for controversies, and those issues that did not have matching points of view were decided by consensus.

## 3. Results

### 3.1. Study Selection

**Figure 1:**
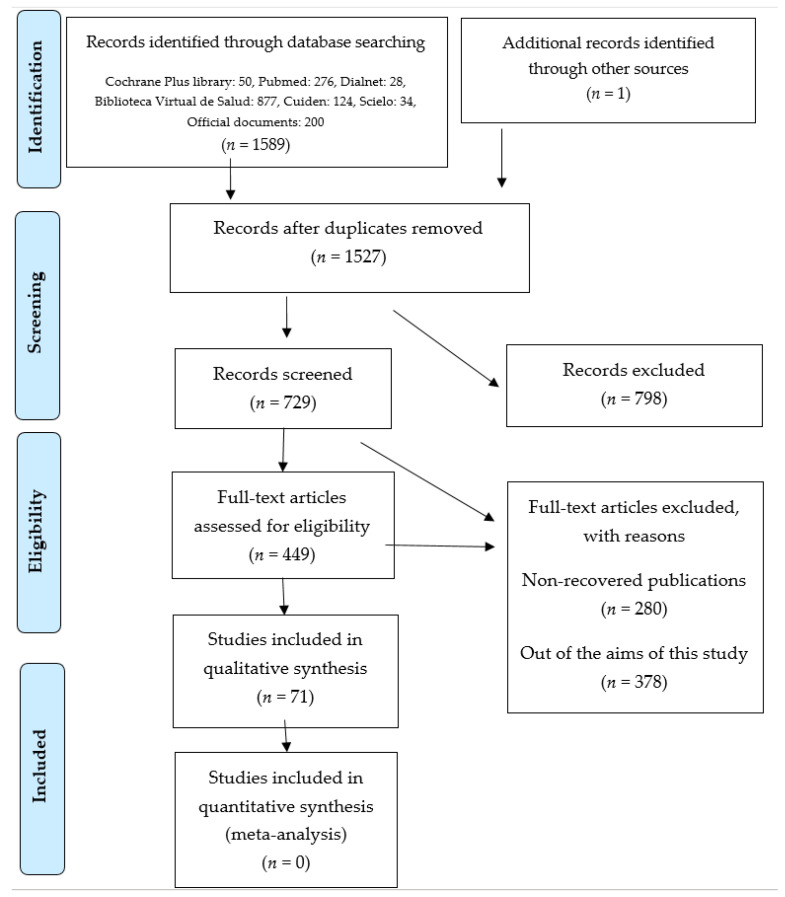
Flowchart of information through the different phases of the systematic review (PRISMA compliant [22]).

A total of 71 items were selected (Figure 1). The number of studies included in the previous version of the systematic review was one. A total of 1589 new studies were identified through databases. A total of 1535 articles were deleted because they did not have access to full text, were duplicated and were inconsistent with the objectives of the study. A total of 16 articles were added through websites.

### 3.2. Characteristics of the studies

The 67 studies and four official documents were included in this review, these studies have been included and detailed in Table S1 [2,3,4,5,6,7,8,9,10,11,12,13,14,15,16,17,18,19,20,21,26,27,28,29,30,31,32,33,34,35,36,37,38,39,40,41,42,43,44,45,46,47,48,49,50,51,52,53,54,55,56,57,58,59,60,61,62,63,64,65,66,67,68,69,70,71].

The selected studies investigated with questionnaires to assess self-concept [2,6,8,10,12,16,27,35,37,38,56,64,65,67,70,71], self-perception [2,3,4,7,8,11,13,14,26,28,29,30,31,32,34,36,38,39,40,42,43,44,45,46,47,48,49,50,51,52,53,57,60,66,68,69], physical activity [2,9,10,15,17,18,55,65] and lifestyle [3,4,5,6,9,14,19,20,21,33,54,58,59,61,62,63] among adolescents.

Studies were conducted in Spain (*n* = 30) [2,4,9,13,15,16,17,18,19,20,21,26,29,35,37,38,39,53,55,56,57,59,61,62,63,65,67,68,71], Colombia (*n* = 5) [5,7,8,36,64], Mexico (*n* = 3) [12,52,54], Cuba (*n* = 1) [11], Argentina (*n* = 2) [10,40], Brazil (*n* = 4) [6,33,44,45], Peru (*n* = 3) [3,31,32], Chile (*n* = 2) [28,60], Uruguay (*n* = 1) [14], USA (*n* = 2) [30,66], Malaysia (*n* = 1) [42], England (*n* = 1) [43], Macedonia (*n* = 1) [46], China (*n* = 1) [51], India (*n* = 2) [48,49] and Indonesia (*n* = 1) [50].

A total of 60 studies employed a case and control design [2,3,4,5,6,7,8,9,10,11,12,13,14,15,16,17,19,21,26,28,29,30,32,33,34,35,36,37,38,39,40,42,44,45,46,47,48,49,50,51,52,53,54,55,56,57,58,59,60,61,62,63,64,65,66,67,68,71], two were clinical trials [18,20], two were cohort studies [31,43] and three were reviews [27,69,70].

A total of six studies were multinational in nature and were conducted with participants from different countries [27,34,47,58,69,70].

### 3.3. Risk of Bias in Individual Studies

The analysis performed with the CASPe [23] critical reading tool is shown in Table S1. The lowest score was 8/11 for clinical trials, case and controls, and cohort studies [6,7,8,9,10,11,12,13,14,17,33,56,61] and 8/10 for systematic reviews [27,69].

### 3.4. Results of Individual Studies

These are grouped into areas of interest according to the data list: self-concept questionnaires, self-perception questionnaires, physical activity questionnaires and lifestyle questionnaires.

#### 3.4.1. Self-Concept Questionnaires

Questionnaires exploring this area identified in the search are the Rosenberg Self-Esteem Scale, the Coopersmith Self-Esteem Inventory for Adults, the Adolescent Coping Scale (ACS) by Frydenberg and Lewis and the Harter’s Self-Perception Profile for Adolescents.

The Rosenberg Self-Esteem Scale (RSES) is the most widely used questionnaire worldwide [7,8,16,26,27]. It was designed by Morris Rosenberg in 1965 and introduced to the world in his book *Society and the Adolescent Self-Image*. It is used to measure people’s self-esteem by detecting, by means of a two-dimensional scale, positive self-esteem (self-confidence or personal satisfaction) and negative self-esteem (self-contempt or personal devaluation) [8]. It is composed of 10 items: five items exploring positive self-esteem and the other five exploring negative self-esteem. The self-confidence/personal satisfaction dimension addresses aspects related to feeling competent in different aspects of life, and the self-contempt/personal devaluation dimension uses pejorative terms associated with self-sympathy [7]. It is assessed on a Likert-type scale from 1 to 4 points, with 4 being the highest score. Negative self-esteem items are classified in the opposite direction. With respect to the total score, 30 to 40 points indicate high self-esteem, this is considered as positive and normal self-esteem for the person; 26 to 29 points refer to intermediate self-esteem, meaning the individual does not have serious self-esteem problems, but it would be ideal to improve it; less than or equal to 25 points is considered low self-esteem, these are usually subjects with significant problems regarding their self-esteem [7,8,16,26,27,28,29,30,31]. The reliability of this questionnaire is derived from the factor analysis and the reliability defined by Cronbach’s alpha, which is 0.85 and 0.88 [26].

The Self-Esteem Inventory-Adult, created by Stanley Coopersmith in 1967, measures a subject’s attitudes in the personal, family and social areas. It is an instrument used for people over 16 years of age. It consists of 25 items distributed in three areas: personal (13 questions), social (6 questions), and the family area (6 questions). To obtain the total score, the number of items answered in correct form must be added together. Subjects scoring between 0 and 24 points have a low level of self-esteem, from 25 to 49 points have a medium-low level of self-esteem, from 50 to 74 points have a medium-high level of self-esteem, and from 75 to 100 points have a high level of self-esteem [6]. The internal consistency of this questionnaire is 0.79 [6].

The Adolescent Coping Scale (ACS) was developed by Frydenberg and Lewis in 1997. It assesses the coping strategies of adolescents aged 12–18 years. However, it could be used for people older than this age. It consists of 80 items, of which 79 are closed-ended and one is an open-ended item. The 80 items are grouped into 18 subscales which in turn are grouped into three different coping styles: coping in relation to others, subscales: seeking social support, social action, seeking spiritual support, and seeking professional help; productive coping or coping aimed at solving the problem, subscales: focus on solving the problem, work hard and achieve, seek to belong, focus on the positive, seek relaxing diversions, and physical recreation; and non-productive coping, subscales: worrying, investing in close friends, wishful thinking, lack of cope, tension reduction, ignoring the problem, self-blame, keeping to oneself. The first 79 items are assessed on a five-point Likert-type scale. The total score is obtained by adding the points for each item. If there is a blank answer, it is scored with an average value of 3. It is understood that applying this value implies less distortion of the results. However, if there are two or more blank items, they should not be scored. Once the scores have been obtained, they are transferred to a profile sheet to obtain the degree of use of the coping strategies. This is indicated on a scale of: 20–29 strategy not used, 30–49 strategy rarely used, 50–69 strategy sometimes used, 70–89 strategy often used, 90–105 strategy very often used. The response to item 80 allows for a qualitative study of other strategies used by the subject that are not collected in the questionnaire. The internal consistency of this instrument is 0.8032. The psychometric adaptation of this scale was developed by Beatriz Canessa on 1236 schoolchildren aged between 14 and 17 years, demonstrating that it is a reliable instrument [32].

The Self-Perception Profile for Adolescents was developed by Harter in 1988. It assesses global self-esteem and self-concept through specific domains. It consists of nine subscales with five items each: physical appearance, romantic appeal, close friendship, social competence, behavioural conduct, scholastic competence, job competence, athletic competence, global self-worth. The adolescent must choose one and state the degree of agreement with that selection. Each item presents four options, with scores ranging from 1 to 4; the higher the score, the higher the perceived competence. The internal consistency of this questionnaire is between 0.75 and 0.93 points [10].

#### 3.4.2. Self-Perception Questionnaires

Questionnaires exploring this area include self-perception and self-perception in relation to health. Those identified in the search are the Goldberg General Health Questionnaire (GHQ), the Harris Self-Concept Scale, the Beck Inventory, Antonovsky’s Sense of Coherence Scale 13 (SOC-13), and Cooper, Taylor and Fairburn’s Body Shape Questionnaire (BSQ).

The General Health Questionnaire (GHQ) was designed by Goldberg in 1972 [4,33,34,35]. It is a test that measures patients’ self-perceived health in two sub-dimensions: psychological well-being and social functioning/coping. It detects non-psychotic psychiatric disorders and measures the person’s mental health in the last 6 months. It identifies two types of problems: the inability to perform normal or adaptive daily tasks, and the manifestation of minor psychopathological symptoms and maladaptive behaviours at personal and social levels. It explores four areas: depression, anxiety, social dysfunction and hypochondriasis [36]. It was initially used in primary care but also in the general population and focused on the psychological components that identify negative health [4,33,34,35,37]. Its first version consisted of 60 items, but over time it was refined and the number of items was reduced with abbreviated versions of 36, 30, 28, 20 and 12 items [36,38,39,40]. The latest version, 12 items, was developed in 1988 by Goldberg and Williams [37,39]. It consists of a two-dimensional structure that assesses depression and social dysfunction [40]. This version has been translated and adapted into 38 languages. Because of its brevity and psychometric characteristics, it is one of the most widely used screening instruments in the world. It is widely used in national health surveys in different countries [32,33,34,35,36,37,39,41,42,43,44,45,46,47,48,49,50,51]. In the Spanish population, its psychometric properties have been analysed in adolescents and postpartum women [39]. There are several studies that indicate the existence of various dimensions within the questionnaire, such as depression/anxiety, social dysfunction, coping strategies, self-esteem and stress [38]. Thus, it has become one of the most widely used instruments for rapid screening of psychological health conditions in the population [38,40]. It consists of 12 items that are assessed on a Likert-type scale from 1 to 4 points. The total score is calculated by adding the scores for each item. As the score increases, the level of mental health decreases [32,33,34,35,37,40]. The internal consistency of this questionnaire is 0.76 [39].

The Piers Harris Self-Concept Scale is a questionnaire, adapted by Cardenal and Fierro, which assesses the individual’s perception of themselves and their own assessment of some aspects of their way of being and behaviour. It is specific for schoolchildren aged between 7 and 12 years. It consists of 80 items formulated with simple sentences and dichotomous answers (yes/no). It is divided into the following dimensions: behavioural (18 items assessing problematic behaviours), intellectual (17 items reflecting self-assessment of academic and school tasks), physical (12 items measuring physical characteristics, leadership, and ability), lack of anxiety (12 items assessing emotions related to concern, nervousness, altered mood, sadness, or fear), social or popularity (12 items focusing on the assessment of their relationships, popularity, and acceptance among their peer group), happiness-life satisfaction (nine items reflecting a general feeling of being happy and satisfied with life), and general (80 items measuring self-perception of physical attributes, behaviour patterns, social relationships, academic performance, emotions, and life satisfaction). High scores would indicate a positive self-concept except for the anxiety subscale, where higher scores indicate lower levels of anxiety. The internal consistency of this questionnaire is 0.89 [9].

The Beck Inventory created in 1983 by the founder of cognitive therapy, Aaron T. Beck. It is a widely used instrument that assesses cognitive symptoms related to depressive states. It can be used from the age of 13. It assesses symptoms of anxiety and/or depression such as hopelessness, irritability, guilt, negative feelings, fatigue, weight loss and loss of sexual interest. It consists of 21 items in which the participant must choose the statement that comes closest to how they have felt in recent weeks. It is evaluated on a Likert-type scale from 0 to 3 points. To obtain the final score, the results must be added up: from 0 to 13 points, the subject does not suffer from depression; from 14 to 19 points, mild depression; from 20 to 28 points, moderate depression; and from 29 to 63 points, severe depression [52]. The Spanish adaptation of this scale shows data on the reliability and validity of the instrument. In the study by Jesús Sanz et al., the participants ranged in age from 18 to 68 years and the internal consistency was high: 0.89 [53].

The Sense of Coherence Scale 13 (SOC-13) created by Antonovsky in 1993. It assesses the dimensions of a person’s ability to perceive the effects of their actions on the environment. It assesses comprehensibility (five items that evaluate the ability to control and manage emotions and thoughts as well as to understand other people); manageability (four items that measure the feeling that a person has to be able to face challenges; related to self-efficacy and competence); and meaningfulness (4 items that measure the value that the subject brings to an act regardless of how it occurs) [38]. It measures why some people become ill while others remain healthy when suffering stressful situations. It is based on positive health development. This scale has been widely used in people over 70 years of age, although to a lesser extent it has also been tested in young university students. There are two versions of the scale, one with 29 items and another reduced version consisting of 13 questions, with a Likert-type response from 1 point (always) to 7 points (never). The result allows the calculation of the mean score for each dimension and the overall mean score to obtain the SOC; an overall score above the mean indicates high SOC [38,54]. The internal consistency of this questionnaire is 0.80 [38].

The Body Shape Questionnaire (BSQ) was created in 1987 by Cooper, Taylor and Fairburn. It was adapted and validated in Spanish by Raich et al. in 1996. It measures dissatisfaction with one’s own body, fear of becoming fat, self-devaluation and physical appearance, desire to lose weight, and avoidance of situations that draw attention to physical appearance in society. It consists of 34 items that are scored on a Likert-type scale from 1 to 6 points. Possible responses are never, rarely, sometimes, often, very often and always. Possible scores: <81 no concern; 81 to 110 mild concern; 111 to 140 moderate concern; and >140 extreme concern. The internal consistency of this instrument is between 0.95 and 0.97 [3].

#### 3.4.3. Physical Activity Questionnaires

Questionnaires exploring this area that were identified in the search are the PAQ-A adolescent activity questionnaire; the Physical Self-Perception Profile by Fox and Corbin; the Physical Self-Concept Questionnaire (CAF, for its acronym in Spanish) by Goñi, Ruiz de Azúa and Rodríguez; the Measurement of the Intention to be Physically Active Scale (MIFA, for its acronym in Spanish) by Moreno and Cervera; and the Children’s Attraction to Physical Activity Questionnaire (CAPA) by Brustad.

The PAQ-A (Physical Activity Questionnaire for Adolescents) assesses the physical activity that the adolescent performed in the last 7 days in their free time, during physical education classes, as well as at different times during the last days of school and during the weekend. The last two questions assess the level of physical activity performed during the week and the amount of physical activity performed each day. It allows to understand at what time of the day and week adolescents are most active. It is made up of 9 questions that assess aspects of physical exercise by means of a 5-point Likert-type scale used in the first eight questions that establishes a graduation regarding the level of physical activity (from less to more) of the adolescent. The last question allows finding out whether or not the adolescent was ill or if there were any circumstances that prevented them from doing physical exercise that week. As a result, a score of 1 to 5 points is obtained, allowing a graduation of the adolescent’s level of physical exercise to be established [5,55]. The PAQ-A questionnaire is one of the most widely used questionnaires on adolescents. It is easy to complete in 10 to 15 min. Several studies using the PAQ-A questionnaire have found associations with indicators of adiposity, bone mineral content, heart rate variability and psychological indicators, such as sports competence, body satisfaction and anxiety. The internal consistency of this questionnaire ranges from 0.77 to 0.84 [55].

Physical Self-Perception Profile is a self-report designed by Fox and Corbin in 1989 for the assessment of physical self-perception. It includes three levels: upper (self-esteem), intermediate (physical self-perception), and lower (physical attractiveness, sport competence, physical strength and physical condition). It consists of 30 items with six items for each of its five subscales: physical self-esteem, sport competence, body attractiveness, physical strength and physical condition. Subjects must describe themselves with a response format designed to eliminate social-desirability bias. It consists of two opposing statements (one positive and one negative) showing two groups of young people with opposing self-perceptions in different aspects. It requires the individual to decide which of these two groups of young people they are more similar to and the degree of similarity with these two groups. It is scored on a Likert-type scale from 1 to 4, where 1 indicates the choice of totally like them in a negative statement and 4 indicates the choice of totally like them in a positive statement. The internal consistency is 0.83 [16].

The Physical Self-Concept Questionnaire (CAF, Spanish version acronym) was created by Goñi, Ruiz de Azúa, and Rodríguez in 2006. It assesses physical self-concept and has been applied in different contexts showing a high level of validity and reliability in high school, university, and adult students. It is made up of 36 items, 20 of which are written directly and 16, inversely. They are divided into 6 dimensions or subscales: general physical self-concept, general self-concept, physical ability, physical condition, physical attractiveness, and strength. Each of the six dimensions is composed of 6 items. Three levels are established: the highest level corresponds to the general self-concept; the intermediate level to the general physical self-concept; and the lowest level to the subdomains of the physical self-concept (physical condition, sport competence, physical attractiveness and strength). It is assessed on a 5-point Likert-type scale where 1 corresponds to a high degree of disagreement and 5 to strongly agree. The scale is reversed for indirect items [4,56]. The internal consistency of this questionnaire is 0.92 [56].

The Measurement of the Intention to be Physically Active (MIFA, Spanish version acronym) scale is a derivative version of the Intention to be Physically Active questionnaire by Heins, Müür, and Koka in 2004. Moreno and Cervello in 2007 adapted this first version to Spanish and called it MIFA. The purpose of this questionnaire is to find out whether physical self-concept has a significant influence on the intention to be physically active. It assesses adherence to physical exercise, motivational climate, autonomy support, basic psychological needs and types of motivation, among others. It has been used in different studies among young athletes. It is made up of five items that measure the subject’s intention to be physically active after passing through different educational institutions. It is assessed on a Likert-type scale from 1 (strongly disagree) to 5 (strongly agree). There is an adaptation of this scale for people who are at university. The Measurement of the Intention to be Physically Active in the University Context (MIFAU) scale was developed by Moreno et al. in 2007. It consists of the 5 items of the MIFA questionnaire, where items 2 and 3 have been modified. It is assessed using a Likert-type scale from 1 to 5 points. The internal consistency is 0.94 [15].

The Children’s Attraction to Physical Activity Questionnaire (CAPA) is a questionnaire that assesses the level of attraction to physical activity (PA). Created in 1993 by Brustad, it was adapted in 2009 by Rose et al. It consists of 4 dimensions, including 18 items: factor 1, enjoyment of PA and sports; factor 2, enjoyment of PA; factor 3, enjoyment of vigorous PA; and factor 4, importance of physical exercise. Responses are scored on a Likert-type scale with values ranging from never, scored as 1, to 5, which corresponds to always. The internal consistency of this questionnaire is 0.94 [17].

#### 3.4.4. Lifestyle Questionnaires

The questionnaires exploring this area identified in the search are: WHO’s Health Behaviour in School-aged Children (HBSC); Diener’s Satisfaction with Life Scale; the Kidscreen-52 questionnaire used to measure Health Related Quality of Life (HRQoL) from the EU Kidscreen project; the MeDiet questionnaire from the PREDIMED study; Berra’s KidMED questionnaire; Walker and Hill Polerecky’s Health Promoting Lifestyle Profile (HPLP II); and Walker, Sechrist, and Pender’s Health-Promoting Lifestyle Profile (HPLP II).

The Health Behaviour in School-aged Children (HBSC) is an international study carried out by the World Health Organization (WHO). It is based on the health-related behaviours of school children. Its initiative started in Finland, Norway and England in 1982 and since then, several editions have been carried out in 2002, 2006, 2010, 2014 and the last one in 2018, which was published in 2020. The aim of this study is to gain an in-depth understanding of the lifestyles of schoolchildren and to analyse their evolution. Each new edition is joined by new countries and in the 2018 edition, 48 countries were taking part. Spain has been involved in this project since 1986. The aim of this study is to obtain a global vision of the lifestyle habits of adolescents and to create tools that enable the promotion of health in this population group. The adolescents surveyed range in age from 11 to 18 years. The questionnaire explores the following variables: socio-demographic, food and diet, oral hygiene, hours of sleep, physical activity and sedentary behaviours, risky consumption, sexual behaviour, injuries, family context, peers and free time, school context, neighbourhood, health and psychological adjustment, and socio-economic inequalities [57,58]. The internal consistency of this questionnaire is 0.90 [58].

The Satisfaction with Life Scale was developed by Diener, Emmons, Larsen, and Griffin in 1985. It measures life satisfaction by eliciting cognitive aspects of well-being. It has been widely used in the university population, obtaining a high level of reliability, although it can also be used in adults. Its measurement is obtained by means of five positive items. Responses are scored on a Likert-type scale with seven possible answers. A score equal to 1 indicates strongly disagree and scores equal to 7 would correspond to strongly agree. Scores: 30–35 highly satisfied; 25–29 satisfied; 20–24 slightly satisfied; 15–19 slightly below average in life satisfaction; 10–14 dissatisfied; and 5–9 very dissatisfied. The internal consistency of this instrument is between 0.79 and 0.893. The Spanish adaptation of the 1–5 Likert-type version was applied to university students in order to obtain an estimate of its reliability and evidence of its validity [59].

The Kidscreen-52 questionnaire is used to measure Health-Related Quality of Life (HRQoL) in children and adolescents (aged 8–18 years). There are three Kidscreen questionnaires translated into several languages: the Kidscreen-52 is the long version, covering 10 dimensions of HRQoL; the Kidscreen-27 is the short version, with 5 HRQoL dimensions; and finally, the Kidscreen-10 is the global measurement of HRQoL. These instruments can be used internationally for European health measurement. They measure the young person’s quality of physical, social and mental well-being. These questionnaires belong to the Kidscreen project, which was funded during the years 2001 and 2004 by the European Commission within the “Quality of Life and Management of Living Resources”. It assesses 10 dimensions of HRQoL, with a total of 52 items: physical well-being (five questions); psychological well-being (six questions); mood and emotions (seven questions); self-perception (five questions); autonomy (five questions); relationship with parents and family life (six questions); financial resources (three questions); friends and social support (six questions); school environment (six questions); and social acceptance (three questions). A Likert-type scale with five response levels is used. The internal consistency of this questionnaire is 0.94 [60].

MeDiet-PREDIMED is a questionnaire on adherence to the Mediterranean diet that was carried out as part of the PREDIMED study. The aim of this study was to identify the dietary pattern, physical exercise, and chronic diseases of the population. MeDiet consists of 14 questions on the consumption of the main food groups of the Mediterranean diet. The scores obtained are grouped into four categories: high adherence (12–14 points); intermediate adherence (8–11 points); low adherence (5–7 points); and very low adherence (<5 points) [61]. The internal consistency of this test is 0.92 [62]. Ana M. Benitez et al. have applied this questionnaire in adolescents in Extremadura, a Spanish region, aged 18–26 years, who showed low adherence to the Mediterranean diet [61].

The Kidmed Questionnaire assesses adherence to the Mediterranean diet as a prototype of a healthy diet. It was developed by Serra et al. between 1998–2000. It consists of 16 questions to be answered with yes or no. It is scored on a Likert scale ranging from 0 (minimum adherence) to 12 (maximum adherence). Different categories are established according to the score: from 8 to 12, optimal Mediterranean diet (high adherence); from 4 to 7, need to improve diet (intermediate adherence); and from 0 to 3, very low-quality diet (low adherence). The internal consistency is 0.70 [5].

The Health Promoting Lifestyle Profile (HPLP II) was designed in 1996 by Walker and Hill Polerecky. This questionnaire aims to profile individuals who seek their own well-being. That is, they pay attention to their health, education and exercise. It is applicable to any age range. It consists of 52 items distributed in six dimensions: health responsibility, physical activity, nutrition, spiritual growth, interpersonal relations and stress management. The items are rated on a Likert-type scale from 1 to 4 (from never to routinely). Internal consistency is between 0.86 and 0.93 [63]. Laguado et al. applied this instrument to young university students with a mean age of 21, where she observed less healthy lifestyle habits than expected [64]. Serrano et al. have adapted it to Spanish for the working population, linking lifestyle with occupational health [63].

The Health-Promoting Lifestyle Profile (HPLP II) created by Walker, Sechrist, and Pender in 1987, assesses lifestyle. It consists of 52 Likert-type questions ranging from 1 point (never) to 4 (routinely). It has six dimensions: health responsibility, physical activity, nutrition, spiritual growth, interpersonal relations and stress management. The sum of the scores of each item ranges from 52 to 208 points and allows to obtain the health promoting lifestyle (HPLS) score. The internal consistency of this instrument is between 0.75 and 0.86 [54].

### 3.5. Synthesis Results

Table S1 presents the results per selected item. Table S2 uses the COSMIN [25] rating for each selected questionnaire based on the analysis of the results of the individual papers.

### 3.6. Publication Bias

Papers that did not exceed a score of 8 in CASPe [23] were eliminated to avoid bias in the results of this review. Table S1 shows the degree of recommendation evaluated with the GRADE system [24].

## 4. Discussion

The main result of this study is the identification of the four questionnaires that emerge from the rest due to their optimal characteristics for each of the areas of study.

In the self-concept questionnaires, the Rosenberg Self-Esteem Scale is ideal for assessment. There are several reasons for this: it is the most widely used questionnaire worldwide. Its creation in 1965 makes it the first questionnaire to assess adolescent self-esteem. It makes it possible to differentiate between positive and negative self-esteem and has also been widely used over time, proving that it is reliable and valid. In addition, it allows a greater comparison of results at the global level [10]. Although the Self-Esteem Inventory-Adult and the ACS could be applied to young people, they are not as specific as the Rosenberg Self-Esteem Scale. In addition, they require a longer time to complete. In the case of ACS, it does not specifically measure the adolescent’s self-concept but the coping strategies they have [6,7,8,16,26,27,32]. In conclusion, the Rosenberg Self-Esteem Scale is the most widely used questionnaire worldwide to detect positive and negative self-esteem. Its use in the adolescent population makes it possible to know the self-esteem of the subject and thus establish corrective measures to prevent the development of common psychiatric disorders [7,8,16,26,27,65,66,67].

Among the questionnaires that assess self-perception, the GHQ-12 stands out above the rest. It is the questionnaire that was first created in 1972. Several versions have been created, from the first version with 60 items to the most recent version with 12 sections. This shows that, with the passage of time and its use in various studies, it has been perfected. It has been translated and adapted into 38 languages. For this reason, it is one of the most widely used instruments in the world and is widely used in national health surveys in different countries. It is able to explore, in only 12 items, the individual’s self-perception of health as well as psychological well-being, depression, anxiety, social inadequacy and hypochondriasis [4,32,33,34,35,36,37,38,39,40]. If we compare it with the other questionnaires evaluated in this systematic review, we find that there are more extensive tools to evaluate anxiety and depression, including the Beck Inventory [52]. Other questionnaires, such as the SOC-13 Scale, measure the behaviours that the adolescent has about the environment but do not include his or her self-perception [37,54]. In conclusion, the GHQ-12 has been perfected over time. It has been used in multiple studies in order to detect self-perception of health. It is a dynamic questionnaire, easy to complete and widely used [4,27,28,29,30,31,32,33,34,35,36,37,38,39,40,68].

Questionnaires assessing physical activity are diverse. The PAQ-A adolescent activity questionnaire stands out from the rest. This instrument assesses the physical activity of the young person in the last week. Consisting of nine questions, it is one of the most widely used questionnaires on adolescents. Moreover, it is easy to complete in 10 to 15 min [5,55]. Other tests evaluated in this systematic review, such as the Physical Self-Perception Profile, are not as complete as the PAQ-A. It is because they value how the individual perceives him/herself physically but not the intensity, duration and physical activity that young people perform [16]. With the CAF, something similar happens, since it allows us to know the physical self-concept but not his or her activity [3,57]. This is also similar for the rest of the questionnaires evaluated, although they evaluate adherence to physical activity, they focus on the individual’s physical self-concept, dissatisfaction with body image and self-assessment [3,15,17]. For these reasons, the PAQ-A is the instrument of choice, by numerous national and international studies, for the evaluation of the physical exercise performed by adolescents [5,55,69].

Finally, there are several questionnaires that assess lifestyle. After compiling a list of tools, the HBSC instrument stands out. It is a study conducted by the WHO and was launched in 1982. It has undergone several updates over the years. With each update, more and more countries have joined the study, reaching 48 by the time of the 2018 edition. It provides a comprehensive overview of adolescents’ lifestyle habits and helps create tools for health promotion. It also allows comparison of lifestyles of adolescents from different countries [57]. Other questionnaires, such as the Life Satisfaction Scale, do not obtain such a global vision of the adolescent since they only evaluate the university population [4]. The rest of the tools described in this systematic review are not as specific as HBSC since they focus on measuring adherence to certain diets, such as MeDiet PREDIMED or Kidmed [5,61]. Although the HPLP-II and CEVII instruments are widely used and could compete with the HBSC study, they do not evaluate parameters that are measured with it. Estimating sociodemographic conditions, feeding, hours of sleep, school and family context, among others, is necessary to determine the life habits of adolescents [58,64]. In addition, the HBSC study has numerous updates in which more countries are participating in each edition. It allows us to have a global vision of the habits in the lives of adolescents of diverse sociodemographic characteristics [57,58].

This systematic review, which compiles the instruments most commonly used in the assessment of self-concept, self-perception, physical exercise, and lifestyle in adolescents, is considered necessary. Despite the high level of education and health prevention in developed countries, obesity, sedentary lifestyles and chronic diseases are on the rise. Investing in studies that seek to understand the adolescent is essential to generate tools that enable the correct approach to be taken at this stage. Establishing corrective measures based on the results provided by the questionnaires is essential in the modification and prevention of behaviours that are harmful to health [2,3,4,5,6,7,8,9,70,71].

Therefore, questionnaires are considered the fastest and most reliable way to obtain information. They must be drafted in a coherent and organised way, sequenced and structured through prior planning that allows their proper use. This study allows us to establish which are the optimal instruments in the integral assessment of the adolescent. In this way, corrective measures can be established that will favour the transition of the young person into the adult stage. Acting on the lifestyle will modify behaviours harmful to the physical and mental health of the adolescent and involves the prevention of chronic non-communicable diseases. It is considered essential to seek new tools for the comprehensive assessment of adolescents since the number of young people with anxiety, depression and overweight problems continues to increase in developed countries. This research group believes that by knowing the appropriate evaluation tools on the self-concept, self-esteem, exercise and lifestyle of adolescents, changes can be made in young people. It is thought that they are able to identify or request help, if required, in the acquisition of healthy lifestyle habits and correct mental health.

### Limitations

This systematic review was conditioned by the validity and reliability of the data provided in the selected scientific articles, as well as the characteristics of the population group under study and the lack of a consensus on the ideal questionnaire to assess self-concept, self-perception, physical exercise and lifestyle as a whole. If further studies with similar objectives were to be conducted, this review could provide the appropriate selection of instruments.

## 5. Conclusions

Questionnaires are therefore considered to be the quickest, most reliable, and valid way of obtaining information. They must be written in a coherent and organised way, sequenced and structured through prior planning that allows them to be used appropriately. Thus, this study enables the establishment of optimal instruments for comprehensive assessment of adolescents.

## Figures and Tables

**Table 1:** Search strategy.

Date of Search	Database	DECS-MeSH Combination	Selection/Results
6 November 2019	BVS	Ejercicio and autopercepción	6/783
6 November 2019	BVS	Estilo de vida and autopercepción	5/94
13 December 2019	Cochrane	Adolescente and estilo de vida	4/10
13 December 2019	Cochrane	Adolescente and autoestima	3/9
20 December 2019	Cochrane	Adolescente and actividad física	5/31
2 January 2020	Cuiden	Autopercepción and cuestionario	2/72
2 January 2020	Cuiden	Autoconcepto and cuestionario	3/21
10 January 2020	Cuiden	Actividad física and adolescentes and cuestionario	5/31
1 July 2020	Scielo	Ejercicio and autoestima	4/34
30 December 2020	Dialnet	Self-concept and questionnaire	4/28
1 July 2020	PubMed	Self-perception and questionnaire	7/47
13 January 2021	PubMed	Exercise and self-concept and questionnaire	5/23
13 January 2021	PubMed	Self-concept and questionnaire and teenager	5/53
10 January 2020	Medline	Exercise and self-perception and teenager	4/53
02 January 2020	Medline	Self-concept and questionnaire	6/100
29 October 2021	Ministerio de Sanidad, Consumo y Bienestar social	Estilo de vida and cuestionario	2/200

Search limits: open access articles with full text available, articles published in the last 5 years, studies in Spanish, English and Portuguese.

## Data Availability

All data are available within this article and its artwork.

## Supplementary Materials

The following are available online at https://www.mdpi.com/article/10.3390/children9010091/s1, Figure S1: PRISMA checklist; Table S1: Articles selected in the search as a source of definitive evidence. CASPe score and GRADE degree of recommendation; Table S2: Assessment of the Cosmin instruments.
