# Supplementary material for: Questionnaires Assessing Adolescents’ Self-Concept, Self-Perception, Physical Activity and Lifestyle: A Systematic Review
Source: Children (Basel). 2022 Jan 10;9(1):91. doi: 10.3390/children9010091 (PMC8774997; doi:10.3390/children9010091)
Supplement: Supplementary file 1 [file children-09-00091-s001.zip › Table S1 (new).pdf]

Table S1. Articles selected in the search as a source of definitive evidence. CASPe score and GRADE degree of recommendation

Ref. num. in the manuscript	Bibliographic reference	Abstract	Decision	CASPe score	GRADE
2	Borrego Balsalobre FJ, López Sánchez G, Díaz Suárez A. Influencia de la condición física en el autoconcepto de un conjunto de adolescentes del municipio de Alcantarilla. Cuader Psicol del Dep [Internet]. 2013 [cited 6 Nov 2019]; 12 (1): 57-62. Available at: https://bit.ly/2z0TReJ	The aim of this study is to establish the normative physical fitness values of Spanish adolescents and to relate them to their self-concept. It is a pilot study with a sample of 53 adolescents aged 14-15 years. As a result, it was observed that students who engaged in physical exercise had better self-perception and self-concept.	This article would make it possible to check whether self-concept, self-perception, and self-esteem improve with physical exercise. In this way, physical activity could be encouraged to improve not only physical but also mental health.	Case and control study 11/11	Moderate
3	Luna Alfaro R, Neira Esquivel R, Román Paredes E, Luna Muñoz C. Autopercepción de la imagen corporal y hábitos alimenticios en estudiantes universitarios, Arequipa 2012. Rev Fac Med [Internet]. 2015 [cited 20 Dec 2019]; 15 (3): 4-10. Available at: https://bit.ly/2Z35lJw	The article determines the self-perception of body image and its relationship with eating habits in Medicine students. The sample consisted of 261 students. The BSQ questionnaire was used to measure body dissatisfaction and a Likert-type questionnaire to determine eating habits. As a result, the higher the perception of body image, the lower the healthy eating habits and vice versa.	The study allows to compare Medicine students with other adolescents and helps us to understand their self-perception and lifestyle.	Case and control study 9/11	Moderate
4	Videra García A, Reigal Garrido R. Autoconcepto físico, percepción de salud y satisfacción vital en una muestra de adolescentes. Anal. Psicol [Internet]. 2013 [cited 20 Dec 2019]; 29(1): 141-147. Available at: https://bit.ly/2P3bLIC	The purpose of this study is to examine the relationships between physical self-concept, health perception, and life satisfaction in adolescents. A total of 1648 adolescents participated. The instruments used are the Physical Self-Concept Questionnaire (CAF, for its acronym in Spanish), the Goldberg General Health Questionnaire (GHQ) and the Satisfaction with Life Scale (SWLS). As a result, a relationship between physical attractiveness and health perception is observed as variables that have a relationship with life satisfaction.	The present study would provide data on adolescents' self-concept, self-perception, and lifestyle. And, in addition, the use of questionnaires for their assessment as a reliable tool.	Case and control study 9/11	Moderate
5	Carrillo HA, Ramírez Vélez R. Adherence to the Mediterranean diet in a simple of Colombian schoolchildren: an evaluation of the psychometric properties of the KIDMED questionnaire. Nutr Hosp. 2019 [cited 30 Dec 2020]; 1(1): 1-25. Available at: https://dx.doi.org/10.20960/nh.02760	The aim of this study was to assess the validity, reliability, and factor structure of the KIDMED questionnaire in a group of children and adolescents from Colombia. 167 school children took part in the study. It showed moderate reliability and reproducibility values, 0.79.	This publication allows us to know the nutritional habits of adolescents with different characteristics from Spanish adolescents.	Case and control study 9/11	Moderate
6	Silva Fhon JR, Ayala Caire M, Valério Mendonça Júnior JA, Partezani Rodríguez RA. Estilo de vida asociado a autoestima y variables demográficas en estudiantes de enfermería. Rev Cient Cienc de la Salud [Internet]. 2016 [cited 6 Nov 2019]; 9(2):	This study validates the association between self-esteem, lifestyle, and demographic variables in Nursing students. The sample consisted of 183 participants. The Demographic Profile Instrument, Self-Esteem Inventory and Lifestyle Instrument were used. As a result, it was observed that 41.5% indicated having intermediate-high self-esteem and 53.6% a healthy	It allows us to know if there is an association between self-esteem and the healthy lifestyle in university adolescents. For this reason, the present study	Case and control study 8/11	Moderate

	72-78. Available at: https://doi.org/10.17162/rccs.v9i2.406	lifestyle. Therefore, there was an association between both variables.	would serve as a reference for this systematic review.		
7	Cogollo Z, Campo Arias A, Herazo E. Escala de Rosenberg para autoestima: consistencia interna y dimensionalidad en estudiantes de Cartagena, Colombia. Psychologia Avanc Disciplina. 2015 [cited 20 Dec 2019]; 9 (2); 61-71. Available at: https://bit.ly/2Z3P7Uq	Cogollo Z, et al. studied the Rosenberg Self-Esteem Scale in a total of 951 high school students aged 10-15 years. As a result, it was shown that the Rosenberg test has a high internal consistency and an acceptable factor solution. However, the authors argue that the Rosenberg Self-Esteem Scale should be considered as two scales, "self-confidence" and "self-contempt".	This article is applicable to the study as it analyses the internal consistency of the Rosenberg Self-Esteem Scale.	Case and control study 8/11	Moderate
8	Ceballos Ospino GA, Paba Barbosa C, Suescún J, Celina Oviedo H, Herazo E, Campo Arias A. Validez y dimensionalidad de la escala de autoestima de Rosenberg en estudiantes universitarios. Pens Psicol [Internet]. 2017 [cited 10 Jan 2020]; 15(2): 29-39. doi:10.11144/Javerianacali.PPSI15-2.vdea	Ceballos Ospino G.A, et al. aimed to know the internal consistency of the Rosenberg scale to assess the self-esteem of a sample of 1341 students, aged 18 to 30 years. As a result, two dimensions were observed: positive self-esteem and negative self-esteem, which behave as distinct scales.	This study is useful as it allows us to assess the Rosenberg Self-Esteem Scale, to see its reliability and dimensions.	Case and control study 8/11	Moderate
9	Gálvez Casas A, Rodríguez García PL, Rosa Guillamón A, García Cantó E, Pérez Soto JJ, Tarraga Marcos L. et al. Relación entre el estatus de peso corporal y el autoconcepto en escolares. Nutr Hosp [Internet]. 2015 [cited 10 Jan 2020]; 31 (2): 730-736. Available at: https://dx.doi.org/10.3305/nh.2015.31.2.8467	Gálvez Casas A, et al. comment that body weight is an element related to different health parameters. To demonstrate this, the relationship between body weight and self-concept was evaluated in a sample of 216 schoolchildren. BMI and the Piers-Harris Self-Concept Scale were used. The results showed an association between BMI, self-concept, and life satisfaction.	This article allows us to know if there is a direct relationship between the development of physical activity and self-concept. It helps us understand the lifestyle of adolescents.	Case and control study 8/11	Moderate
10	Daniela Calero A, Fernanda Molina M. Más allá de la cultura: validación de un modelo multidimensional de autoconcepto en adolescentes argentinos. Escritos de Psicología [Internet].2016 [cited 10 Jan 2020];9(1):33-41. Available at: https://dx.doi.org/10.5231/psy.writ.2016.1201	The study explains that self-concept and self-esteem are key constructs in explaining adolescent development. The aim was to statistically contrast the structure of the SPPA questionnaire in the adolescent population of the Autonomous City of Buenos Aires, Argentina. The sample consisted of 467 adolescents. The article allowed refining the adaptation of the SPPA questionnaire for its use in adolescents.	This study makes some significant contributions. On the one hand, it shows the importance of self-concept and self-esteem as they affect people's physical and mental health, and allows us to know in which areas it is important to focus interventions in adolescents.	Case and control study 8/11	Moderate
11	Peláez E, Débora Acosta L, Delia Carrizo E. Factores asociados a la autopercepción de salud en adultos mayores. Rev Cubana Salud Pública [Internet]. 2015 [cited 6 Nov 2019]; 41(4):638-648. Available at: https://bit.ly/2YMYOHu	This article assesses health status in older adults through self-perception of health and self-perception. As a result, it is observed that 60.1 % had a positive self-perception of health. In contrast, negative self-perceived health was related to being female, having no health coverage, having no income or income below USD 250, needing help, having more than one chronic	The study carried out by Peláez E., et al. serves to compare the self-perception of health of adolescents with that of older adults and to observe whether there	Case and control study 8/11	Moderate

		disease, low or medium educational level, and dissatisfaction with life. This indicates that self-perceived health is useful for measuring the health status of a population, as it is related to the presence of chronic diseases. Additionally, help received and life satisfaction were related to self-perceived health.	are significant differences in age, personal maturity, and sex.		
12	Escamilla Cruz S, Córdoba Ávila MA, Campos Castolo EM. Autopercepción de competencias profesionales de alumnos de la Licenciatura en Enfermería. Rev Conamed [Internet]. 2012 [cited 6 Nov 2019]; 17 (2): 67-75. Available at: https://bit.ly/2z7iWoJ	Escamilla Cruz S, et al. evaluate the self-perception of Health Sciences undergraduates who will face the work environment and current social demands as future professionals. The study was carried out on a total sample of 137 students using a 32-item instrument. As a result, it was observed that Nursing students self-perceive their level of competence as adequate for their training.	This study allows us to find out whether Nursing students perceive an adequate level in their studies. However, it would not serve to evaluate the objective of this article.	Case and control study 8/11	Moderate
13	Fernández Ayuso D, Del Campo Cazallas C, Fernández Ayuso RM, Pérez Olmo JL, Morillo Rodríguez J, Matías Pompa B. Relación entre la autopercepción y autoeficacia para el desarrollo de competencias en soporte vital en entorno de simulación clínica de alta fidelidad. Educ Med [Internet]. 2017 [cited 13 Dec 2019]; 1(1):1-7. Available at: https://doi.org/10.1016/j.edumed.2017.03.030	This article examines whether there is a relationship between self-perception and self-efficacy for critical patient care in the training of Nursing students. As a result, an increase in self-perception for the development of competencies associated with a critical situation was observed, with variations between both groups in relation to self-perception and self-confidence. For this reason, it is advisable to recommend clinical simulation for the training of students in life support.	Although the article provides information on the relationship between self-perception and self-efficacy for the care of critically ill patients in the training of Nursing students, it does not fit the methodology or objectives of this systematic review.	Case and control study 8/11	Moderate
14	De Souza Cruz MC, Mariscal Crespo MI. Competencias y entorno clínico de aprendizaje en enfermería: autopercepción de estudiantes avanzados de Uruguay. Rev Enf Glob [Internet]. 2016 [cited 13 Dec 2019]: 15(41): 121-134. Available at: https://bit.ly/33xewp5	This article describes the perception of competence level by Nursing students in relation to the practical learning environment. The sample consisted of 33 students. The Nurse Competence Scale and the Practice Environment Scale-Nursing Work Index questionnaires were used. As a result, it was found that the students had a very good level of competence. Self-assessment, through instruments such as the NCS, is a fundamental tool for the evaluation of the competences achieved.	Although this article seems interesting from the point of view of nursing competencies, it does not provide representative data on self-perception, self-concept, lifestyle, and physical exercise of university adolescents.	Case and control study 8/11	Moderate
15	Moreno JA, Moreno R, Cervelló E. El autoconcepto físico como predictor de la intención de ser físicamente activo. Psicol y Salud [Internet]. 2013 [cited 6 Nov 2019]; 17(2): 261-267. Available at: https://bit.ly/2TuVlHN	Physical self-concept was found to be a predictor of being physically active. The sample consisted of 988 students who were administered the Physical Self-Perception Profile (PSPP) and the Measurement of the Intention to be Physically Active (MIFA, for its acronym in Spanish) scales. The results revealed that the intention to be physically active is predicted by the physical self-concept. The authors consider the promotion of physical-sports practice to be important.	Moreno J.A., et al. compare whether physical self-concept is a predictor of being physically active. Thus, this study would be useful to compare whether adolescents who are physically active have a better self-concept and self-perception than the rest. The promotion of physical	Case and control study 9/11	Moderate

			activity in adolescents would be justified.		
16	Luis Atienza F, Balaguer I, Moreno Y, Fox KR. El perfil de autopercepción física: propiedades psicométricas de la versión española y análisis de la estructura jerárquica de las autopercepciones físicas. Psicothema [Internet]. 2004 [cited 13 Dec 2019]; 16 (3): 461-67. Available at: https://bit.ly/3bI2bT6	The main objective of this study was to study the psychometric properties of the Physical Self-Perception Profile and to analyse the validity of the hierarchical structure of physical self-perceptions.<br>Two samples of adolescents from the Valencian Community were collected for this study. The first sample consisted of 474 subjects (15-17 years old), and the second sample consisted of 51 subjects (14-19 years old). To obtain the responses, the self-report Physical Self-Perception Profile (PSPP) was used, consisting of 30 items, as well as the Rosenberg Self-Esteem Scale.	The study seems interesting because it allows us to analyse whether physical exercise significantly influences people's self-esteem.	Case and control study 10/11	Moderate
17	Ruiz Ariza A, Ruiz JR, Torre Cruz M, Latorre Román P, Martínez López E. Influencia del nivel de atracción hacia la actividad física en el rendimiento académico de los adolescentes. Rev Latinoam Psicol [Internet]. 2016 [cited 6 Nov 2019]; 48: 42-50. Available at: https://doi.org/10.1016/j.rlp.2015.09.005	This study aims to find out the level of attraction towards physical activity and academic performance, and its association. A total of 1009 adolescents were recruited. The Children's Attraction to Physical Activity Questionnaire and subject grades were used. Analyses showed that vigorous physical activity is the main factor of attraction related, in girls, to better grades. In boys, enjoyment of games and sports and vigorous physical activity did not influence their academic performance. It is suggested that the relationship between physical activity and academic performance may be related to different academic performance outcomes according to sex.	Ruiz Ariza A., et al. report that physical activity and academic performance are related to each other, with sportswomen being those who score better. Therefore, it would be possible to find out whether physical exercise has a significant influence on adolescents.	Case and control study 8/11	Moderate
18	Iglesias Martínez B, Olaya Velázquez I, Gómez Castro MJ. Prevalencia de realización y prescripción de ejercicio físico en pacientes diagnosticados de ansiedad y depresión. Aten Primaria [Internet]. 2015 [citad 13 Dec 2019]; 47(7): 428-37. Available at: https://doi.org/10.1016/j.aprim.2014.10.003	Iglesias Martínez B, et al. have conducted a descriptive, observational and cross-sectional study with participants aged 18 to 75 years diagnosed with anxiety and/or depression. They aim to estimate the prevalence of the practice and prescription of physical exercise in this sample.	This study would serve to test whether or not participants with low self-esteem and self-concept engage in physical exercise. However, the sample would not be representative, as it includes people with a mean age of 55 years.	Clinical prediction rules 9/11	Moderate
19	Rizo Baeza MM, González Brauer NG, Cortés E. Calidad de la dieta y estilos de vida en estudiantes de ciencias de la salud. Nutr Hosp [Internet]. 2014 [cited 6 Nov 2019]; 29(1):153-157. Available at: https://dx.doi.org/10.3305/nh.2014.29.1.6761	This article evaluates the eating habits of students of the University of Alicante of the degrees of Human Nutrition and Dietetics, and Nursing. The sample consisted of a total of 184 subjects (96 from Human Nutrition and Dietetics and 88 from Nursing). BMI and 24-hour food consumption were calculated. As a result, the majority of the students analysed have a normal weight. No differences in diet quality were found between the two degrees. There is also a higher consumption of saturated fat and lower consumption of polyunsaturated fat than	The article by Rizo Baeza M.M., et al. would help to compare the lifestyle and level of knowledge that Health Sciences adolescents have with respect to the rest and whether they are able to apply it in their daily lives.	Case and control study 10/11	Moderate

		recommended. Nutrition students exercise more than Nursing students, although they sleep less and spend more time in front of the computer and TV. Therefore, it is concluded that knowledge of nutrition does not influence healthy eating and lifestyle choices.	It serves to reflect on the educational model and whether it is possible to implement the knowledge acquired.		
20	Sánchez Socarrás V, Aguilar Martínez A, Vaqué Crusellas C, Milá Villarroel R, González Rivas F. Diseño y validación de un cuestionario para evaluar el nivel de conocimientos generales en trastornos del comportamiento alimentario en estudiantes de ciencias de la salud. Aten Primaria [Internet]. 2016 [citad 13 Dec 2019]; 48(7): 468-78. Available at: https://dx.doi.org/10.3305/nh.2014.30.1.7451	This article is composed of a total sample of 2400 adolescents aged between 12 and 17 years from the Autonomous Region of Andalusia. Antolín Suárez et al. aim to assess values within the framework of promoting positive adolescent development. The instrument used was the Positive Adolescent Development Value Scale (EV-DPA, for its acronym in Spanish) which assesses the importance that young people have on a set of values involved in PAD.<br>This is an observational, longitudinal and prospective study to validate a questionnaire to assess the level of knowledge about eating disorders in university students. The sample consisted of 140 Health Sciences students (53 women and 87 men). As a result of the study, a 10-question instrument was obtained to assess the level of knowledge of eating disorders among Health Sciences students.	In this systematic review we aim to describe the correct lifestyle that university adolescents should have. In this way, this study would help us to identify the knowledge that Health Sciences students have about eating disorders, not about their lifestyle.	Clinical trial 10/11	Moderate
21	Mera Gallego R, Mera Galleo I, Fornos Pérez JA, García Rodríguez P, Fernández Cordeiro M, Rodríguez Reneda Á. Análisis de hábitos nutricionales y actividad física de adolescentes escolarizados. RIVACANGAS. Rev Esp Nutr Comunitaria. 2017 [cited 20 Dec 2019]; 23 (1): 1-12. Available at: https://bit.ly/34fmQdj	The aim of this study was to get to know the lifestyle of 630 students in compulsory secondary education. Questions were asked about their diet and physical activity. The KIDMED questionnaire (adherence to the Mediterranean diet) and the PAQ-A questionnaire (physical activity) were used. The results showed that eating habits are better among girls and worsen with age. In addition, moderate adherence to the Mediterranean diet and moderate physical activity were found, being higher among boys and at a younger age.	This study is representative because it analyses the lifestyle of a sample of adolescents using two reliable questionnaires applicable to this study.	Case and control study 11/11	Moderate
26	Martín Albo J, Nuñez JL, Navarro JG, Grijalvo F. The Rosenberg Self-Esteem Scale: Translation and Validation in University Students. The Spanish Journal of Psychology. 2007 [cited 20 Dec 2019]; 10 (2): 458-67. doi:10.1017/S1138741600006727	This research translates into Spanish and analyses the Rosenberg Self-Esteem Scale (RSES) in a sample of university students. As a result, positive and strong correlations are observed between self-esteem and five dimensions of self-concept. Furthermore, the scale has shown satisfactory levels of internal consistency and temporal stability over a four-week period. Finally, significant sex differences were obtained. These results support the use of the RSES to assess self-esteem in the university educational context.	The research carried out by Martín Albo J., et al. would provide significant data as it would allow us to use the Rosenberg Self-Esteem Scale in older university adolescents.	Case and control study 9/11	Moderate
27	Brea M. Informe de la interpretación de las escalas CPS, Rosenberg y Grit y propuestas de revisión para mejorar la confiabilidad. The World Bank. 2011 [cited 20 Dec 2019]; 1 (1): 1-36. Available at: https://bit.ly/2KW6Jsv	In this study, the Personal and Social Competence Scale (PSC), the Rosenberg test and the GRIT test are defined. The values of the answers are described, a report for the improvement of each questionnaire is elaborated, the reliability of the questionnaires is analysed, and some corrections are made.	This report is useful for comparing self-esteem questionnaires, checking for corrections, and analysing their usefulness and reliability.	Systematic review 8/10	Moderate

28	Rojas Barahona CA, Zegers B, Förster CE. La escala de autoestima de Rosenberg: Validación para Chile en una muestra de jóvenes adultos, adultos y adultos mayores. Rev Med Chil [Internet]. 2009 [cited 1 July 2020];137(6):791-800. Available at: http://dx.doi.org/10.4067/S0034-98872009000600009	Rojas Barahona, et al. aimed to determine the reliability and validity of the RSES in a sample of Chilean adults. The instrument was administered to 473 subjects living in the Metropolitan Region of Santiago. The variables under study were: age, sex, educational level, and income. The RSES meets the criteria of validity and reliability of a quality instrument to measure self-esteem in Chilean citizens. Its behaviour is similar to that expected by Rosenberg in the general population.	It helps to compare the results of the validity and reliability of the RSES in populations with different socio-cultural characteristics.	Case and control study 11/11	High
29	Vázquez Morejón AJ, García Bóveda RJ, Morejón Jiménez RV. Escala de autoestima de Rosenberg: fiabilidad y validez en población clínica española. Apunt Psico [Internet]. 2004 [cited 1 July 2020]; 22 (2): 247-55. Available at: https://bit.ly/2GEfjNW	It studies the reliability and validity of the Rosenberg self-esteem scale (RSES), in the version adapted to Spanish. The sample consisted of 533 mental health patients. The results confirm high internal consistency and satisfactory reliability. The ease of application of this test and its acceptable psychometric characteristics constitute an important support for its use in clinical contexts.	It provides information on the Spanish version of the RSES, its reliability and validity in mental health patients.	Case and control study 11/11	High
30	González Rivera JA, Pagán Torres OM. Validación y análisis de la dimensionalidad de la Escala de Autoestima de Rosenberg en mujeres puertorriqueñas: ¿unifactorial o bifactorial?. Apuntes de Psicología [Internet]. 2017 [cited 2 July 2020]: 35(3), 169-178. Available at: https://bit.ly/3mRLeef	The aim of this study was to examine the reliability, validity, and dimensional structure of the RSES in a total of 340 Puerto Rican women. The results confirmed that the scale has a bifactor structure. These dimensions are positive self-esteem and negative self-esteem.	The choice of this article is necessary because the RSES is a valid, reliable, and widely used tool for the assessment of self-esteem in the general population.	Case and control study 10/11	High
31	Ventura León J, Caycho Rodríguez T, Barboza Palomino M. Evidencias psicométricas de la escala de autoestima de Rosenberg en adolescentes limeños. Interam Jourl of Psyco [Internet]. 2018 [cited 2 July 2020]; 52 (1): 44-60. Available at: https://doi.org/10.30849/rip/ijp.v52i1.363	Instrumental study in two samples, the first made up of 450 (226 females and 224 males) and the second of 481 (225 females and 256 males) Peruvian adolescents belonging to public and private educational institutions in Metropolitan Lima. The RSES has empirical evidence of validity, reliability, and non-invariant functioning of some items.	This article was chosen because it demonstrates the internal consistency of the RSES, its validity and reliability. It is very useful as it allows comparison of the results with other studies using this scale worldwide.	Cohort study 10/11	High
32	Canessa B. Adaptación psicométrica de las Escalas de Afrontamiento para Adolescentes de Frydenberg y Lewis en un grupo de escolares de Lima metropolitana. Univ Lima [Internet]. 2002 [cited 13 Jan 2021]; 5(1):191-233. Available at: https://bit.ly/3rEvJck	This scale is shown to be reliable and to have construct validity, as item-test correlations on each of the scales obtained significant values.	It is a useful study for this systematic review as it assesses the reliability and validity of a coping scale for adolescents. This provides insight into how young people behave and cope with their problems.	Case and control study 10/11	High

33	Gonçalves Câmara S, Castellá Sarriera J, Carlotto MS. Predictores de conductas sexuales de riesgo entre adolescentes. Interamerican Journal of Psychology [Internet]. 2007 [cited 13 Dec 2019]; 41(2): 161-66. Available at: https://bit.ly/3smenjG	This article assesses predictors of sexual risk behaviour. It analyses socio-demographic, psychosocial, and health variables. The sample consisted of 389 students. The results showed that young men had had the highest number of sexual partners in the last year, the highest frequency of alcohol consumption, and the lowest index of psychological well-being. There is a strong social pressure on the expected behaviour for a male, with a totally different representation for males and females.	This study is useful for assessing adolescent risk behaviours and lifestyle.	Case and control study 8/11	Moderate
34	Brabete AC. El cuestionario de Salud General de 12 items (GHQ-12): estudio de traducción y adaptación de la versión rumana. Rev Iberoam Diag Eval. 2014 [cited 20 Dec 2019]; 1 (37): 11-29. Available at: https://bit.ly/33u7eSR	Brabete A.C. analyses the psychometric properties of the General Health Questionnaire (GHQ-12) in a sample of 806 participants aged 16 to 78 years. The results of the study confirm that the GHQ-12 is an effective tool for assessing psychological well-being and detecting non-psychotic psychiatric problems.	It is considered useful for this study as it evaluates the GHQ-12 questionnaire and confirms its validity for assessing psychological well-being.	Case and control study 10/11	Moderate
35	Sanchez López P, Saavedra San Roman AI, Dresch V, Garcia Quintans L, Rodrigo Holgado I. Women are more at risk of por mental health: mental health of Spanish nurses measured by the GHQ12. Womens's Health Bull. 2016[cited 13 Dec 2019]; 3(2). doi: 10.17795/whb-29603	This article analyses mental health in a group of nurses in a hospital in Madrid (Spain) to assess whether there are differences between the groups. In addition, there is an analysis of whether there are differences by sex. The GHQ-12 instrument is used. As a result, nurses scored worse on mental health than the general population. Women performed worse than men. Mental health in nurses is related to age, self-perceived health, job stress, and job satisfaction. Variables related to mental health in nurses are job stress and rest/sleep.	This article is interesting because in addition to using the GHQ-12 questionnaire, it assesses the mental health of the nursing profession. Its results could help to influence psychological health in the stage of adolescence and the training of this profession.	Case and control study 9/11	Moderate
36	Villa G. IC, Zuluaga Arboleda C, Restrepo Roldán LF. Propiedades psicométricas del Cuestionario de Salud General de Goldberg GHQ-12 en una institución hospitalaria de la ciudad de Medellín. Avanc Psicol Latinoam. 2013 [cited 20 Dec 2019]; 31 (3): 532-45. Available at: https://bit.ly/2YM4HEK	This study evaluates the properties of the GHQ-12 in a group of 85 patients. The overall results are in line with previous standardisation studies where the GHQ-12 questionnaire shows adequate psychometric characteristics.	The article described has an impact on this study as it evaluates the GHQ-12 questionnaire and shows its reliability.	Case and control study 10/11	Moderate
37	B. Rocha K, Pérez K, Rodríguez Sanz M, Borrel C, E. Obiols J. Propiedades psicosométricas y valores normativos del General Health Questionnaire (GHQ-12) en población general española. Int J Clin Health Psychol.2011 [cited 13 Dec 2019];11(1):125-39. Available at: https://bit.ly/2H4KBuA	This is a study that uses data from the Spanish National Health Survey to analyse the GHQ 12. The sample consists of 29,476 people. The results show that the GHQ-12 can be used as a unidimensional screening instrument and the most appropriate score in this case is the GHQ score.	This article is interesting as it analyses the reliability of the GHQ12 questionnaire.	Case and control study 10/11	Moderate
38	Fernández Martínez ME, Mayo Chamorro L, García Mata MA, Liébana Presa C, Fernández García D, Vázquez Casares AM. Sentido de	This study compares the reliability of the measurement instruments used (Sense of Coherence Scale and the Goldberg General Health Questionnaire) as well as the relationship of the	The choice of the article seems interesting as it would allow us to compare	Case and control study	Moderate

	coherencia y salud percibida en alumnos universitarios de ciencias de la salud. ANESM [Internet]. 2016 [cited 13 Dec 2019]; 1(1). Available at: https://bit.ly/2vOeMUv	sense of coherence with psychological health in students of Health Sciences.<br>A total of 297 students from the University School of Health Sciences of the Ponferrada Campus of the University of León took part in the study. 79.7% were women and 20.3% were men, aged 18-47 years.	the psychological health state of Health Sciences students by obtaining information on their self-perception and self-concept.	11/11	
39	Sánchez López MP, Dresch V. The 12-Item General Health Questionnaire (GHQ-12): reliability, external validity and factor structure in the Spanish population. Psicothema. 2008 [cited 13 Dec 2019]; 20(4):839-43. Available at: https://bit.ly/2KLmlyy	The aim of this study was to analyse the internal consistency and the external and structural validity of the 12-item General Health Questionnaire (GHQ-12) in the general Spanish population. The sample consisted of 1,001 subjects. As a result, it is observed that the GHQ-12 can be used to assess psychological well-being and detect non-psychotic psychiatric problems.	This article is relevant because it deals with the GHQ12 and analyses its validity. This questionnaire is useful for the development of this study.	Case and control study 11/11	Moderate
40	Moreta Herrera R, López Calle C, Ramos Ramírez M, López Castro J. Estructura factorial y fiabilidad del cuestionario de Salud General de Goldberg (GHQ-12) en universitarios ecuatorianos. Rev Arg Cienc Comp [Internet]. 2018 [cited 10 Jan 2020]; 10 (3): 35-42. Available at:https://doi.org/10.32348/1852.4206.v10.n3.20405	This article evaluates the GHQ-12 by determining its reliability and validity in a sample of 587 university students. As a result, the GHQ-12 showed high validity for the use of mental health assessments in university adolescents.	The article described here has an impact on the study because it analyses the GHQ-12 questionnaire in a sample of university students. Its data can be extrapolated to this review.	Case and control study 10/11	Moderate
41	Quek KF, Low WY, Razack AH, Chua CB, Loh CS. Reliability and Validity of the Malay Version of the General Health Questionnaire (GHQ - 12) Among Urological Patients. Med J Malaysia [Internet]. 2002 [cited 1 July 2020];57(2):169-177. Available at: https://bit.ly/3bsdSgL	The aim of the study was to validate the Malay version of the General Questionnaire (GHQ-12) in patients with psychiatric morbidity secondary to urological disorder. Validity and reliability were studied in patients with lower urinary tract symptoms (LUTS) and patients without LUTS.	This study provides useful information on the reliability and validity of the GHQ-12 questionnaire in a population diagnosed with a psychiatric disorder secondary to a urological procedure.	Case and control study 10/11	High
42	Quek KF, Low WY, Razack AH, Loh CS. Reliability and validity of the General Health Questionnaire (GHQ-12) among urological patients: a Malaysian study. Psychiatry Clin Neurosci [Internet]. 2001 [cited 1 July 2020];55(5):509-513. Available at: https://bit.ly/3jIKxlf	The present study aimed to validate the English version of the General Health Questionnaire (GHQ - 12) in urological patients. Validity and reliability were studied in patients with lower urinary tract symptoms (LUTS) and patients without LUTS. Reliability was assessed using the test-retest method and internal consistency was assessed using Cronbach's alpha.	The article allows us to observe the validity and reliability of the GHQ-12 questionnaire in an adult population. In this way, the importance of this questionnaire and its wide use in different environments and populations can be confirmed.	Case and control study 10/11	High
43	Hankins M. The reliability of the twelve-item General Health Questionnaire (GHQ-12) under	This study aimed to establish the best-fit model of the GHQ-12 for three scoring methods (Likert, GHQ, and C-GHQ) and to	It allows the GHQ-12 questionnaire to be		High

	realistic assumptions. BMC Public Health [Internet]. 2008 [cited 1 July 2020]; 8 (355): 1-7. Available at: https://doi.org/10.1186/1471-2458-8-355	calculate the degree of measurement error under these more realistic assumptions. The GHQ-12 data were obtained from a 2004 England Health Survey cohort. The best-fit model was unidimensional with response bias on negatively worded items, suggesting that the previous factor structures of the GHQ-12 were artefacts of the method of analysis.	evaluated as a valid and reliable tool for assessing the subject's self-perception.	Cohort study 9/11	
44	Gouveia VV, Barbosa GA, Oliveira Andrade Ed, Carneiro MB. Factorial validity and reliability of the General Health Questionnaire (GHQ-12) in the Brazilian physician population. Cad Saude Publica [Internet]. 2010 [cited 1 July 2020];26(7):1439-1445. Available at: https://doi.org/10.1590/S0102-311X2010000700023	The GHQ-12 is a widely used screening instrument. This study aimed to assess its factorial validity and reliability, and to test one-factor and two-factor models. Participants were 7,512 Brazilian physicians. They answered the GHQ-12 and demographic questions.	This article is suitable for assessing the reliability of the GHQ-12 questionnaire. Although the subjects do not have the same characteristics as those in this study, it allows us to observe the efficacy of this tool in different populations and settings.	Case and control study 9/11	Moderate
45	Gouveia VV, Lima TJ, Gouveia RS, Freires LA, Barbosa LH. Questionário de Saúde Geral (QSG-12): o efeito de itens negativos em sua estrutura fatorial [General Health Questionnaire (GHQ-12): the effect of negative items in its factorial structure]. Cad Saude Publica [Internet]. 2012 [cited 1 July 2020];28(2):375-384. Available at: https://doi.org/10.1590/S0102-311X2012000200016	Although the GHQ-12 is commonly treated as a general index, the definition of its factor structure is a matter of debate. This paper aimed to test this structure by comparing three models: two frequently cited in the literature (one factor and two factors) and a third one, also unified, that controls for response bias by item wording.	The decision to include this article is due to the fact that it analyses the validity and reliability of the GHQ-12. Of the 1180 participants, one group (university students) resembles our sample, so the results of this study may be significant.	Case and control study 9/11	Moderate
46	Petkovska MS, Bojadziev MI, Stefanovska VV. Reliability, Validity and Factor Structure of the 12-Item General Health Questionnaire among General Population. Open Access Maced J Med Sci [Internet]. 2015 [cited 1 July 2020];3(3):478-483. Available at: https://doi.org/10.3889/oamjms.2015.075	Results revealed a higher GHQ score among women than men. Participants from rural areas obtained a lower GHQ-12 score compared to participants from urban areas. The GHQ-12 can be effectively used to assess general psychological well-being and detect non-psychotic psychiatric problems among the Macedonian population.	The article fits the needs of this study as it assesses the GHQ-12 in different areas (urban and rural) of a population with sociocultural characteristics different from Spanish society.	Case and control study 10/11	Moderate
47	Gelaye B, Tadesse MG, Lohsoonthorn V, Lertmeharit S, Pensuksan M.C, Sanchez S.E, et al. Psychometric properties and factor structure of the General Health Questionnaire as a screening tool for anxiety and depressive symptoms in a multi-	To examine the reliability, construct validity, and factor structure of the GHQ-12 in a sample of young African, Asian, and South American university students.	The choice of this article seems interesting as it allows a comparison of the GHQ-12 questionnaire between young adult		High

	national study of young adults. J Affect Disord [Internet]. 2015 [cited 2 July 2020]; (187):197-202. Available at: https://doi.org/10.1016/j.jad.2015.08.045		university students from different geographical areas. Moreover, the results show that it is a valid and reliable tool for the detection of common psychiatric disorders.	Case and control study 11/11	
48	Kashyap GC, Singh SK. Reliability and validity of General Health Questionnaire (GHQ-12) for male tannery workers: a study carried out in Kanpur, India. BMC Psychiatry [Internet]. 2017 [cited 2 July 2020];17(1):102-9. Available at: 10.1186 / s12888-017-1253-y	The findings suggest that the Hindi version of GHQ-12 is a reliable and valid tool for measuring psychological distress in male tannery workers in Kanpur city, India.	It was decided to select this study because it confirms that the GHQ-12 questionnaire is a scale for measuring the psychological well-being of various groups of people in different settings.	Case and control study 10/11	High
49	Qin M, Vlachantoni A, Evandrou M, Falkingham J. General Health Questionnaire-12 reliability, factor structure, and external validity among older adults in India. Indian J Psychiatry [Internet]. 2018 [cited 2 July 2020]; 60(1):56-59. Available at: https://doi.org/10.4103/psychiatry.IndianJPsychiatry_112_17	Analyses data collected as part of the UNFPA project "Building Knowledge Base on Ageing in India (BKPAI)". The BKPAI survey was conducted in 2011 in seven major demographically advanced states of India: Himachal Pradesh, Punjab, West Bengal, Odisha, Maharashtra, Kerala, and Tamil Nadu. A representative community-based sample of 9692 respondents aged 60 years and older was used. The GHQ-12 shows adequate reliability among the older population in India.	This study was chosen because it analyses the structure, validity, and reliability of the GHQ-12. However, the sample analysed is not adolescents.	Case and control study 10/11	High
50	Anjara SG, Bonetto C, Van Bortel T, Brayne C. Using the GHQ-12 to screen for mental health problems among primary care patients: psychometrics and practical considerations. Int J Ment Health Syst [Internet]. 2020 [cited 2 July 2020]; 14:62. Available at: https://doi.org/10.1186/s13033-020-00397-0	The internal consistency of the GHQ-12 was good. The GHQ-12 was found to fit a unidimensional model better when response bias is taken into account. The Indonesian version of the GHQ-12 could be used to assess primary care patients at high risk for mental disorders, albeit with significant false positives if reasonable sensitivity is to be achieved.	This study is useful because it analyses the internal consistency of the GHQ-12, which is valid to ratify its usefulness as a method for detecting non-psychotic psychiatric disorders.	Case and control study 10/11	High
51	Liang Y, Wang L, Yin X. The factor structure of the 12-item General Health Questionnaire (GHQ-12) in young Chinese civil servants. Health Qual Life Outcomes [Internet]. 2016 [cited 2 July 2020];14(1):136-45. Available at: https://doi.org/10.1186/s12955-016-0539-y	This study examines the factor structure of the GHQ-12 in young Chinese civil servants. Respondents include 1051 participants from six cities in eastern China. Results indicate that the GHQ-12 has very good reliability and validity. The Chinese version of the GHQ-12 is suitable for professional groups and can serve as a screening tool to detect anxiety and psychiatric disorders.	This article was chosen because it verifies the validity and reliability of the GHQ-12 questionnaire. Despite the fact that the population does not match the characteristics of the sample, the accuracy of the results seems adequate.	Case and control study 10/11	High

52	Soria Trujano R, Ávila Ramos E, Sandoval Rodríguez MD. Estudio de género sobre depresión y consumo de tabaco y alcohol en estudiantes de carreras del área de la salud y otras áreas. Rev Elect Psicol Iztacala [Internet]. 2015 [cited 13 Dec 2019]; 18(2): 879-903. Available at: https://bit.ly/2MYlVYA	The aim of the present study was to measure the incidence of depression, the frequency of alcohol and other beverage intake, and tobacco use in Health and non-Health students, with comparisons between sex and professions. The sample consisted of 100 students. The Beck Depression Inventory and a Likert-type scale were used. Health risk habits were found in all samples and the absence of depression predominated. For this reason, the authors of the article suggest studying social support and coping with stress.	This is an article that provides significant data for the present study as it measures the lifestyle and the self-perception and self-concept of Health Sciences students.	Case and control study 10/11	Moderate
53	Sanz JG, García Vera MP, Espinosa R, Fortún M, Vázquez Valverde C. Adaptación española del Inventario para la Depresión de Beck-II (BDI-II): 3. Propiedades psicométricas en pacientes con trastornos psicológicos. Clín y Salud [Internet]. 2005 [cited 13 Jan]; 16 (2): 121-142. Available at: https://bit.ly/3l33DGM	The Beck Depression Inventory-II is shown to be a valid instrument for detecting and quantifying depressive symptoms. The reliability coefficient was high (0.89).	This article allows us to compare its results with those of other studies that analyse the subject's self-perception of him/herself. Moreover, it allows us to validate this scale in the Spanish population.	Case and control study 9/11	High
54	Escobar Castellanos B, Cid P, Juvinyà D, Sáez K. Estilo de vida promotor de salud y sentido de coherencia en adultos jóvenes universitarios. Hacia. Prom. Salud [Internet]. 2019 [cited 10 Jan 2020]; 24 (2): 107-122. doi: 10.17151/hpsal.2019.24.2.9	This is a descriptive study of 300 university students in Mexico. The article identifies the health-promoting lifestyle and the relationship with the sense of coherence. It puts into practice the theoretical foundations that can improve the health behaviours of the population.<br>Thus, Health Sciences students (55.2%) had an insufficient health-promoting lifestyle, with statistically significant differences by degree. The overall sense of coherence was high (52.0%), predominantly in men (58.0%) and Health Sciences students (57.2%). As a result, it was evident that a sufficient health-promoting lifestyle promotes a high sense of coherence. Therefore, it is necessary to establish life skills programmes by ensuring better health and well-being and by promoting a sense of coherence.	This article could be used to compare, in subsequent studies with the same methodology, whether Health Sciences students differ from the rest by having information on the behaviours to follow to improve their own health or whether, on the contrary, they have an insufficient promotive lifestyle.	Case and control study 10/11	Moderate
55	Martínez Gómez D, Martínez de Haro V, Pozo T, Welk GJ, Villagra A, Calle ME et al. Fiabilidad y validez del Cuestionario de Actividad Física PAQ-A en adolescentes españoles. Rev Esp Salud Pública. 2009 [cited 20 Dec 2019]; 3 (83): 427-39. Available at: https://bit.ly/34g9Wfd	The purpose of this research was to assess the reliability and validity of the Physical Activity Questionnaire (PAQ-A) questionnaire in 82 Spanish adolescents between 12 and 17 years old. As a result, the PAQ-A shows adequate reliability and reasonable validity to assess physical exercise in Spanish adolescents.	This research is interesting for this study because it determines the reliability of the PAQ-A questionnaire in adolescents.	Case and control study 11/11	Moderate
56	Molero D, Zagalaz Sánchez ML, Cachón Zagalaz J. Estudio comparativo del autoconcepto físico a lo largo del ciclo vital. Rev Psicol Deport [Internet]. 2013 [cited 20 Dec 2019]; 22 (1); 135-142. Available at: https://bit.ly/33vZ8cs	The study by Molero D, et al. examines self-concept across the life course to see if there are statistical differences according to age and sex. They recruited 152 participants who answered the Physical Self-Concept Questionnaire (CAF, for its acronym in Spanish). As a result, physical self-concept gradually improves	This article allows us to find out whether or not physical self-concept improves with respect to age and sex. It would serve to compare adolescents	Case and control study 8/11	Moderate

		with age, with significant differences being found in the female group.	with other stages of the life cycle.		
57	Moreno C, Ramos Valverde P, Rivera F, García Moya I, Jiménez Iglesia A, Sánchez Queija I, et al. Cuestionario HBSC-2014 en España. Ministerio de Sanidad, Servicios Sociales e Igualdad [Internet]. 2016 [cited 20 Dec 2019]. Available at: https://bit.ly/3bD8G9q	This article describes the HBSC questionnaire in Spain. Different sections explain what the questionnaire consists of, the target population, the different items it is composed of, and how it is evaluated.	The study is considered convenient because it describes the HBSC questionnaire in Spain, allowing its access and use.	Case and control study 11/11	High
58	Inchley J, Currie D, Budisavljevic S, Torsheim T, Jåstad A, Cosma A et al. Spotlight on adolescent health and well-being. WHO [Internet]. 2020 [cited 13 Jan 2021]; 1(2): 1-144. Available at: https://.ly/3BOvgZS	Data from the HBSC survey in different European countries are described. It allows a comparison of the lifestyles of adolescents with different cultures and life habits.	It is a valid, reliable, and widely used questionnaire at an international level. This instrument allows us to compare Spanish adolescents with young Europeans, observing the similarities and differences regarding lifestyle.	Case and control study 11/11	High
59	Garrido Muñoz MR, Fernández Borrero MA, Villalba Ruiz EB, Pérez Moreno PJ, Fernández Bellido M. Evidencias de validez de la adaptación al español de la escala de satisfacción con la vida en una muestra de estudiantes universitarios. Met de Enc [Internet]. 2010 [13 Jan 2021]; 12(1):45-62. Available at: https://bit.ly/3l1Ej3L	Life satisfaction is described and the scale is validated in a Spanish university population to obtain an estimate of its reliability and validity.	It allows us to compile a tool tested in the Spanish adolescent population that is valid and reliable in the measurement of lifestyle and life satisfaction.	Case and control study 9/11	Moderate
60	Molina Cartes R, Sepúlveda R, Molina T, Martínez V, González E, Leal I, et al. Calidad de vida relacionada con la salud en adolescentes chilenos, según autopercepción de discapacidad, enfermedad o problemas de salud crónicos. Rev Chil Salud Pública [Internet]. 2014 [cited 10 Jan 2020]; 18(2): 149-160. doi:10.5354/0719-5281.2014.31976	This article is about health-related quality of life. The sample size was 7910 students aged 10-18 years. As a result, it is observed that health-related quality of life is worse in females and older adolescents. It provides a new and useful input for future youth health programmes in the public sector.	This study would help to compare the self-perception of health of Spanish adolescents with that of Chilean adolescents, thus justifying the creation and modification of current youth health programmes.	Case and control study 9/11	Moderate
61	Benítez Benítez AM, Sánchez S, Franco Reynolds L, Bermejo ML, Cubero J. Adhesión a la dieta mediterránea en estudiantes de la Universidad de Extremadura: un recurso en educación para la salud. FEM [Internet]. 2016 [cited 10 Jan 2020]; 19(6): 287-289. Available at: https://bit.ly/33yrId5	It assesses adherence to the Mediterranean diet in Health Sciences students, justifying that education can be an effective and innovative resource within health education. A sample of 600 students who were administered the Mediterranean diet adherence questionnaire (MeDiet-PREDIMED) was analysed. As a result, low scores for adherence to the Mediterranean diet were observed, the highest being those of students of university degrees in Health Sciences. University students of Extremadura do not follow the precepts of the Mediterranean diet, so an intervention is needed in these young people through health	This study makes comparing Health Sciences adolescents with the rest possible, to observe if lifestyle is modified and therefore, if it affects their self-perception and self-concept. It is a useful resource for the present study.	Case and control study 8/11	Moderate

		education. There is a greater adherence to the Mediterranean diet in the Health Sciences degrees.			
62	Salas Salvadó J, Mena Sánchez G. El gran ensayo de campo nutricional PREMIDED. Nutr Clin Med. 2017 [cited 10 Jan 2020]; 11(1):1-8. Available at: doi:10.7400/NCM.2017.11.1.5046	This is a clinical nutrition intervention trial for the primary prevention of cardiovascular disease. Spanish adult women and men were included. It showed that following a Mediterranean diet is effective in the reduction and prevention of cardiovascular diseases.	This work allows us to know what nutritional habits the population should follow in order to have a better quality of life and to reduce diseases derived from an unhealthy lifestyle.	Case and control study 9/11	Moderate
63	Serrano Fernández MJ, Boada Grau J, Vigil Colet A, Gil Ripoll C. Adaptación española de la escala HPLP-II con una muestra de empleados. Univ Psic [Interntet]. 2016 [cited 18 Jan 2021]; 15(4):1-13. Available at: https://doi.org/10.11144/Javeriana.upsy15-4.aeem	The aim of this research was the validity of this scale in Spanish. The results support a four-factor solution, reducing the number of items from 52 to 48, with adequate reliability.	A scale that allows us to know the lifestyle of the Spanish working population is explored. Its results can be compared with other tools that measure lifestyle.	Case and control study 9/11	Moderate
64	Laguado Jaimes E, Gómez Díaz MP. Estilos de vida saludable en estudiantes de enfermería en la universidad cooperativa de Colombia. Rev Hacia la Promoción de Salud [Internet]. 2014 [cited 6 Nov 2019]; 19(1): 68-83. Available at: https://bit.ly/2emuubT	The article determines healthy lifestyles in Nursing students using Nola Pender's HLPII instrument. The sample consisted of 154 students with the predominant sex being female with 85.7%. The participants ranged in age from 16 to 38 years. As a result, it is observed that Nursing students have a series of factors that are not considered healthy by the questionnaire used. There is evidence of risk behaviours that may influence the students' self-perception/self-concept.	This study would allow us to compare Nursing students with the rest of the adolescents and to observe if there are differences between them. Thus, it helps us to know the influential factors in the self-perception and self-concept as well as the lifestyle of adolescents studying Health Sciences.	Case and control study 9/11	Moderate
65	Delgado B, Inglés CJ, García Fernández JM. Social Anxiety and Self Concept in Adolescence. Rev Psicodid [Internet]. 2013 [cited 6 Nov 2019]; 18 (1):179-195. doi:10.1387/RevPsicodidact.6411	Delgado B, et al. analysed the relationships between social anxiety and dimensions of self-concept in a sample of 2,022 Spanish students aged 12 to 16. The Social Phobia and Anxiety Inventory (SPAI) and the Self-Description Questionnaire (SDQ-II) were used for their assessment. The results show that adolescents with social anxiety have low self-esteem. In addition, students with social anxiety are more likely to perceive their relationships with same-sex peers negatively, to consider themselves worse students, less attractive and less athletic, and to be more emotionally unstable than students without social anxiety. The implication of sex and grade level on the relationship between social anxiety and self-concept during adolescence is discussed.	The article "Social Anxiety and Self Concept in Adolescence" allows us to assess whether anxiety influences self-perception, self-concept, and physical exercise. It helps us to understand if there are sex differences and if there is a direct relationship between lifestyle, physical exercise, and self-perception/self-concept.	Case and control study 11/11	Moderate

66	Rosenberg, M. Society and the adolescent self-image. Princeton University Press. 2015 [cited 20 Dec 2019]. Available at: https://bit.ly/2Zx6AoA	The article defines the Rosenberg Self-esteem Scale in adolescents. It studies positive correlates such as self-esteem and self-concept. It also compares social attitudes and how they influence individual self-concept.	This article is necessary because it defines the Rosenberg Self-Esteem Scale and its use in a sample of adolescents.	Case and control study 9/11	Moderate
67	Tomás Sábado J, T. Limonero J. Propiedades psicométricas de la Escala de Autoestima de Rosenberg en una muestra española. Psiquiat. 2008 [cited 10 Jan 2020]. Available at: https://bit.ly/2M3mRN9	This article aims to analyse the psychometric properties of the Spanish version of the Rosenberg Self-Esteem Scale. A sample of 164 Nursing students between 18 and 42 years of age was recruited. A reliability of 0.89 was obtained. The validity and reliability of this tool was confirmed.	It is very useful for comparing the results of this scale in other adolescents with similar characteristics.	Case and control study 11/11	High
68	Tomás JM, Hontangas P, Oliver A, Galiana L, Sancho P. More on the dimensionality of the GHQ-12: competitive confirmatory models. Univers Psych [Internet]. 2019 [cited 2 July 2020]; 18 (1): 1-9. Available at: https://doi.org/10.11144/Javeriana.upsy18-1.mdgc	Additional evidence of the factorial validity of the Spanish version of the GHQ-12 is presented through competitive factor models. Three samples of workers are used. Results show that the multidimensional models had moderate to high inter-factor correlations but not high enough to compromise the discriminant validity of the models. There is a three-factor multidimensional structure underlying the GHQ-12 items.	This article is of great importance because it analyses the structure, reliability, and validity of the GHQ-12 questionnaire in the Spanish population, including adolescents.	Case and control study 10/11	High
69	Moreno X, Huerta M, Albala C. Autopercepción de salud general y mortalidad en adultos mayores. Gac Sanit [Internet]. 2014 [cited 13 Dec 2019]; 28(3):246-252. Available at: https://dx.doi.org/10.1016/j.gaceta.2013.07.006	Moreno X, et al. explore the association between self-perception of general health and mortality in older adults. As a result, six out of seven found a higher risk of dying in people who rated their health as poor. The results therefore support previous findings that negative self-perceived general health predicts mortality.	Although this article is not related to adolescents, it provides data on self-perceived health in older adults by sex that can be compared with the self-perceived health of young people, and allows us to see if there are differences by age.	Systematic review 8/10	Moderate
70	Moreno Mínguez A. Rasgos característicos de la transición a la vida adulta de los jóvenes españoles en el marco comparado europeo. Pensamiento iberoamericano [Internet]. 2008 [cited 13 Dec 2019]; 1(3): 17-46. Available at: https://bit.ly/2SwA2qf	This study aims to compare the transition to adulthood of Spanish and European young people.<br>During the article it is commented that the precariousness of jobs and the delay in acquiring economic and family independence as a result of the so-called globalising and individualising process have an influence.<br>However, young Spaniards are different from the rest. On the one hand, the configuration of the welfare state model, and therefore the policies devised in that context and the labour market; and, on the other hand, the family culture that continues to be a distinctive element of the strategies adopted by young Spaniards.	Moreno Mínguez's article seems useful because it allows us to compare Spanish adolescents with European adolescents and to extrapolate and compare our data with other international studies.	Systematic review 9/10	Moderate

		In short, young Spaniards show a family dependency and a growing precariousness of individualisation.			
71	Antolín Suárez L, Oliva Delgado A, Pertegal Vega MA, López Jiménez AM. Desarrollo y validación de una escala de valores para el desarrollo positivo adolescente. Psicothema [Internet]. 2011 [cited 13 Dec 2019]. 23(1): 153-59. Available at: https://bit.ly/3bI2bT6	Two samples of adolescents from the Valencian Community were collected for this study. The first sample consisted of 474 subjects (15-17 years old), and the second sample consisted of 51 subjects (14-19 years old). To obtain the responses, the self-report Physical Self-Perception Profile (PSPP) was used, consisting of 30 items, as well as the Rosenberg Self-Esteem Scale.<br>This article is composed of a total sample of 2400 adolescents aged between 12 and 17 years from the Autonomous Region of Andalusia. Antolín Suárez et al. aim to assess values within the framework of promoting positive adolescent development. The instrument used was the Positive Adolescent Development Value Scale (EV-DPA, for its acronym in Spanish) which assesses the importance that young people have on a set of values involved in PAD.	The Positive Adolescent Development Value Scale (EV-DPA, for its acronym in Spanish) would allow us to examine the self-perception and self-concept of adolescents. This is why the article is considered relevant to the study.	Case and control study 10/11	Moderate
