# Supplementary material for: Questionnaires Assessing Adolescents’ Self-Concept, Self-Perception, Physical Activity and Lifestyle: A Systematic Review
Source: Children (Basel). 2022 Jan 10;9(1):91. doi: 10.3390/children9010091 (PMC8774997; doi:10.3390/children9010091)
Supplement: Supplementary file 1 [file children-09-00091-s001.zip › Table S2.pdf]

Table S2. Assessment of the Cosmin [25] instruments.

	Self-esteem/self-concept				Self-perception					Physical exercise					Lifestyle						
	RSES[26]	Self-Esteem Inventory for Adults[6]	ACS[33]	Self-Perception Profile for Adolescents[10]	GHQ-12[39]	Piers-Harris Self-Concept Scale[9]	Beck Inventory[51]	SOC 13[38]	BSQ[2]	PAQ-A[55]	Self-Perception Profile[16]	CAF[56]	MIFA[15]	CAPA[17]	HBSC[58]	Satisfaction with Life Scale[3]	Kidscreen 52[60]	MeDiet PREDIMED[62]	Kidmed[5]	HPLP II (1996)	HPLP II (1987)
1. Formulate the objective of the review.	Yes	Yes	Yes	Yes	Yes	Yes	Yes	Yes	Yes	Yes	Yes	Yes	Yes	Yes	Yes	Yes	Yes	Yes	Yes	Yes	Yes
2. Formulate the eligibility criteria.	Yes	Yes	Yes	Yes	Yes	Yes	Yes	Yes	Yes	Yes	Yes	Yes	Yes	Yes	Yes	Yes	Yes	Yes	Yes	Yes	Yes
3. Conduct a bibliographic search.	Yes	Yes	Yes	Yes	Yes	Yes	Yes	Yes	Yes	Yes	Yes	Yes	Yes	Yes	Yes	Yes	Yes	Yes	Yes	Yes	Yes
4. Select abstracts and full-text articles.	Yes	Yes	Yes	Yes	Yes	Yes	Yes	Yes	Yes	Yes	Yes	Yes	Yes	Yes	Yes	Yes	Yes	Yes	Yes	Yes	Yes
5. Extract data on the characteristics of the measuring instruments included and information on feasibility and interpretability.	Yes	Yes	Yes	Yes	Yes	Yes	Yes	Yes	Yes	Yes	Yes	Yes	Yes	Yes	Yes	Yes	Yes	Yes	Yes	Yes	Yes
6. Assess content validity.	Yes	Yes	Yes	Yes	Yes	Yes	Yes	Yes	Yes	Yes	Yes	Yes	Yes	Yes	Yes	Yes	Yes	Yes	Yes	Yes	Yes
7. Assess internal structure.	Yes	No	Yes	Yes	Yes	Yes	Yes	Yes	Yes	Yes	No	No	Yes	No	Yes	Yes	Yes	No	No	Yes	Yes
8. Assess reliability and measurement error	Yes	Yes	No	Yes	Yes	Yes	Yes	Yes	Yes	Yes	Yes	Yes	Yes	Yes	Yes	Yes	Yes	No	No	Yes	Yes
9. Assess other measurement properties (criterion	Yes	Yes	No	No	Yes	No	No	Yes	Yes	Yes	Yes	No	No	No	Yes	No	No	No	Yes	Yes	Yes

validity, hypothesis testing for construct validity, and sensitivity).																					
10. Formulate recommendations.	Yes	Yes	Yes	No	Yes	Yes	No	No	No	Yes	No	No	Yes	Yes	Yes	Yes	Yes	Yes	No	No	No
11. Select a systematic review.	Yes	Yes	Yes	No	Yes	Yes	No	No	No	Yes	No	No	Yes	Yes	Yes	Yes	Yes	Yes	No	No	No
Internal consistency	0.85 0.88	Yes	0.80	0.75 AND 0.93	0.76	0.89	0.74	0.80	0.95 AND 0.97	0.77 AND 0.84	0.83	0.92	Yes	0.94	0.90	0.79 AND 0.89	0.94	0.92	0.70	0.86 AND 0.93	0.75 AND 0.86

RSES: Rosenberg Self-Esteem Scale.

GHQ: General Health Questionnaire.

BSQ: Body Shape Questionnaire.

CAF (Spanish acronym): Physical Self-Concept Questionnaire.

CAPA: Children's Attraction to Physical Activity Questionnaire.

HPLP II (1987): Health Promoting Lifestyle Profile (Walker, Sechrist, and Pender, 1987).

ACS: Adolescent Coping Scale.

SOC-13: Sense of Coherence Scale.

PAQ-A: Physical Activity Questionnaire for Adolescents.

MIFA (Spanish acronym): Measurement of the Intention to be Physically Active.

HBSC: Health Behaviour in School-aged Children.

HPLP II (1996): Health Promoting Lifestyle Profile (Walker and Hill Polerecky, 1996).
